# Atomic-Site Coordination Tuning for Precise CO_2_ Electroconversion

**DOI:** 10.1021/prechem.5c00434

**Published:** 2026-03-09

**Authors:** Tianshang Shan, Gengxian Zhou, Hongpan Rong, Jiatao Zhang

**Affiliations:** † School of Materials Science & Engineering, 47833 Beijing Institute of Technology, Beijing 100081, China; ‡ School of Chemistry and Chemical Engineering, Beijing Key Laboratory of Intelligent Molecular Materials and High-throughput Manufacturing, MIIT Key Laboratory of Medical Molecule Science and Pharmaceutical Engineering, MOE Key Laboratory of Cluster Science, Beijing Institute of Technology, Beijing 100081, China; § Beijing Institute of Technology, Zhuhai 519088, China; ∥ School of Computer Science (National Pilot Software Engineering School), Beijing University of Posts and Telecommunications, Beijing 100876, China; ⊥ Zhongguancun Academy, Beijing 100094, China

**Keywords:** Electrochemical CO_2_ reduction, Carbon neutrality, Coordination environment, Electronic structure, Single-atom site catalysis, Precise synthesis, Structure−activity relationship

## Abstract

Electrochemical carbon dioxide reduction (ECR) presents a promising avenue for achieving carbon neutrality by converting greenhouse gases into high-value fuels and chemicals. However, the advancement of ECR hinges on the precise design and synthesis of catalysts that exhibit high activity, selectivity, and stability. Single-atom site catalysts (SASCs), benefiting from their maximum atom-utilization efficiency, uniform and tunable active sites, and unique electronic properties arising from strong metal–support interactions, have emerged as a powerful platform for investigating the structure–activity relationship of ECR. By tuning coordination environments (e.g., coordination types and numbers) of isolated metal atoms, the electronic structure of metal active sites can be precisely modified for governing the selective synthesis of ECR products. Herein, this review systematically summarizes the precise synthesis strategies, advanced characterization techniques, and structure–activity relationships of SASCs in various coordination environments. Furthermore, the limitations and necessary precautions associated with the current characterization techniques are also discussed. Finally, we outline the future challenges and potential research directions of SASCs in the field of ECR.

## Graphical abstract

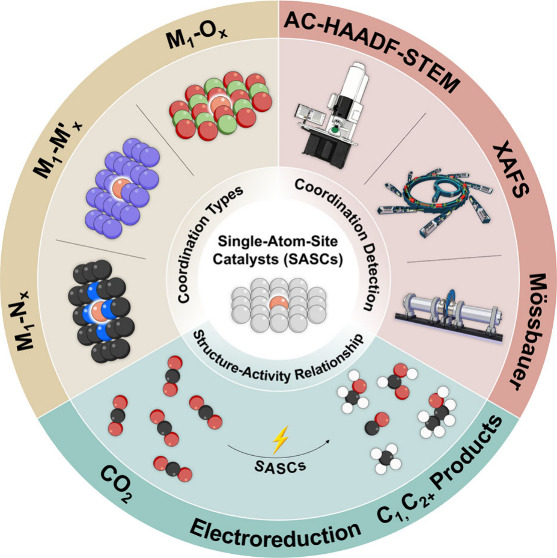

## 1 Introduction

With the rapid acceleration of global industrialization, the massive consumption of fossil fuels has precipitated a drastic surge in carbon dioxide (CO_2_) emissions, triggering severe global environmental crises such as the greenhouse effect and ocean acidification.^1^ Despite the continuous advancement of renewable energy technologies, projections indicate that fossil fuels will continue to dominate the global energy consumption structure until at least 2030, implying that carbon emissions will not be easily mitigated in the short term.^2^ In this background, Carbon Capture, Utilization, and Storage (CCUS) technologies have garnered significant attention for their potential in carbon reduction.^3^ Specifically, the CCUS pathway involving the conversion of CO_2_ into high value-added chemicals offers a promising solution to simultaneously address carbon emissions and energy shortages.^4^ Currently, major CO_2_ conversion technologies include electrocatalysis,^5–12^ thermocatalysis,^13–15^ photocatalysis,^16–20^ and biocoupled catalysis.^21–23^ Among these, electrochemical CO_2_ reduction (ECR) dates back to the 19th century, when the French scientist Royer first achieved the reduction of CO_2_ to formic acid on a zinc electrode in 1870.^24^ Distinguished by its environmental friendliness and sustainability, ECR utilizes clean electricity to drive the direct conversion of CO_2_ into C_1_ (e.g., CO, CH_4_, and HCOOH) and C_2+_ (e.g., C_2_H_4_, C_2_H_5_OH, and CH_3_COOH) value-added carbon-based fuels and chemicals under ambient conditions.^25–27^ Consequently, this field has witnessed rapid development over the past century.^28–37^

Nevertheless, the practical implementation of the ECR faces substantial challenges. The core scientific obstacle lies in the high thermodynamic stability of the CO_2_ molecule, which possesses a strong C=O bond (bond energy: 806 kJ·mol^–1^). Furthermore, the reaction involves complex proton-coupled electron transfer (PCET) processes, resulting in sluggish kinetics and high reaction overpotentials.^38,39^ Simultaneously, the competitive hydrogen evolution reaction (HER) at the cathode often diminishes the faradaic efficiency (FE) of carbon products,^40^ while the high energy barrier for C–C coupling makes the selective regulation of high value-added C_2+_ products particularly difficult.^41^ Therefore, the rational design and preparation of electrocatalysts with high activity, selectivity, and long-term stability are critical for the efficient operation of ECR. In recent years, single-atom site catalysts (SASCs), characterized by near 100% atom utilization efficiency and uniform active sites (defined as an isolated metal atom center coordinated with surrounding heteroatoms), have emerged as frontier materials in the ECR field.^42^ The concept of such catalysts can be traced back to 1925, when Taylor first proposed a surface catalysis theory involving gaseous Ni single-atom active sites;^43^ however, it did not initially attract widespread attention. The field entered a period of rapid expansion in 2011 after Zhang et al. explicitly conceptualized ″single-atom catalysis″ and introduced the M_1_/Support notation.^44^ Since then, Li et al. have established a ″SASCs toolbox″ covering nearly all nonradioactive elements in the periodic table by developing precise synthesis methodologies—such as host–guest strategy,^45–47^ in situ coordination strategy,^48,49^ and high-temperature solid-phase reactions strategy.^50,51^ These advances have significantly propelled the industrialization of SASCs in applications including automotive exhaust purification,^52^ alkane dehydrogenation,^53^ and ECR.^54^ Compared to traditional nanocatalysts (e.g., bulk metals,^55–57^ metal oxides,^58–61^ and carbon materials^62,63^ ), SASCs with unique electronic properties arising from strong metal–support interactions offer a revolutionary platform for elucidating the structure–activity relationship in the field of ECR. By precisely regulating the coordination environment (e.g., coordination types and numbers) of metal active sites^64^ at the atomic scale, SASCs are primarily categorized into M_1_-N_*x*_ (N: nitrogen),^65–67^ M_1_-M’_*x*_ (M’: another metal),^68–70^ and M_1_-O_*x*_ (O: oxygen).^71,72^

In light of these developments, this Review systematically summarizes the precise synthesis strategies for SASCs with distinct coordination structures. Then, we critically evaluate the developments and intrinsic limitations of high-angle annular dark-field scanning transmission electron microscopy (AC-HAADF-STEM),^73^ X-ray absorption fine structure spectroscopy (XAFS)^74^ and Mössbauer spectroscopy^75^ used for SASCs coordination detection. Next, the intrinsic correlations between these coordination structures and the catalytic ECR performance (C_1_ and C_2+_ products) are comprehensively elucidated. Finally, we provide a perspective on the future challenges and potential research directions of SASCs in the field of ECR, aiming to provide a roadmap for the further advancement and practical application.

## 2 Coordination Types of SASCs

Driven by the high surface energy, isolated metal atoms are prone to agglomeration into thermodynamically more stable metal nanoparticles or clusters.^76^ Thus, the anchoring effect provided by the coordinating atoms of the support is vital for achieving an atomic-level dispersion. In view of this, the term ″single-atom site catalysts″ (SASCs) is adopted in preference to ″single-atom catalysts″ (SACs) to emphasize that the intrinsic active sites are not merely the single metal atoms, but rather the ensembles formed by the isolated metal atoms and their surrounding coordinating atoms (e.g., M’,^77–79^ N,^80–82^ O,^72,82,83^ C,^84–86^ and S^87^). Based on the distinction of these coordinating atoms, we herein highlight the specific synthesis strategies for three main types of SASCs (M_1_-N_*x*_, M_1_-M’_*x*_, and M_1_-O_*x*_), offering a clear guide for their design principles.

### 2.1 M_1_-N_*x*_

M_1_-N_*x*_ generally refers to active sites in carbon-based SASCs where a central metal atom coordinates with a varying number of N atoms and other nonmetal heteroatoms (typically ≤ 4, except for lanthanides (>4) mainly due to their larger ionic radii^1,88^ ), which have been widely applied in ECR research.^89–91^ The more electronegative coordination heteroatoms of M_1_-N_*x*_ can create highly localized acceptor-like states near Fermi energy, leading to electron transfer from the metal atoms, which stabilizes the metal centers and effectively inhibits atomic aggregation.^92^ In general, these coordination structures can be categorized into two main groups: symmetric M_1_-N_4_ ^93–95^ and asymmetric M_1_-N_*x*_A_*y*_,^96,97^ where A represents vacancies^98–100^ or nonmetal heteroatoms such as sulfur (S),^101,102^ phosphorus (P),^103,104^ boron (B),^105,106^ O,^107,108^ and chlorine (Cl).^109,110^

To construct these SASCs with diverse coordination structures, the synthesis can be broadly summarized as the strategic combination of metal precursors^111–117^ with substrate precursors like N sources,^118–124^ nonmetal heteroatom sources,^85,89,125^ and C sources.^126–130^ So far, pyrolysis remains the most widely used combination strategy to synthesize SASCs with symmetric M_1_-N_4_ or asymmetric M_1_-N_*x*_A_*y*_ structures. For example, Li et al. used N-doped porous carbon, derived from the high-temperature carbonization of 2-Methylimidazole zinc salt (ZIF-8), as a support. By precisely adsorbing Ni^2+^ followed by thermal activation at varying temperatures (400–1200 °C), they successfully achieved fine-tuning of the Ni–N_*x*_ coordination structure. Quantitative extended XAFS (EXAFS) fitting revealed that the coordination number of Ni–N_*x*_ decreased from ∼4.06 at 900 °C to ∼3.54 at 1200 °C, directly proving that the temperature can drive the transition from symmetric Ni–N_4_ to asymmetric Ni–N_3_ (Figure 1a).^131^ Besides, He et al. presynthesized NiO nanosheets as self-templates. Then, in an atmosphere of C/N species generated by urea decomposition, NiO was reduced to metallic Ni cores, while an N-doped carbon layer formed simultaneously on the surface. Finally, Ni atoms were anchored into the defects of the N-doped carbon layer via strong Lewis acid–base interactions, thereby forming SASCs with high-density asymmetric Ni–N_2_ single-atom sites (Figure 1b).^132^ Certainly, asymmetric M_1_-N_*x*_A_*y*_ SASCs can also be prepared via other methods. For instance, Dong et al. applied microwave treatment (1000 W, 5 s) to Cu^2+^-adsorbed amino-functionalized graphene nanosheets (AGNs) to rapidly synthesize Cu–N_3_ SASCs, as evidenced by the Cu–N coordination number of ∼ 2.8 from EXAFS fitting (Figure 1c).^133^

**Figure 1:**
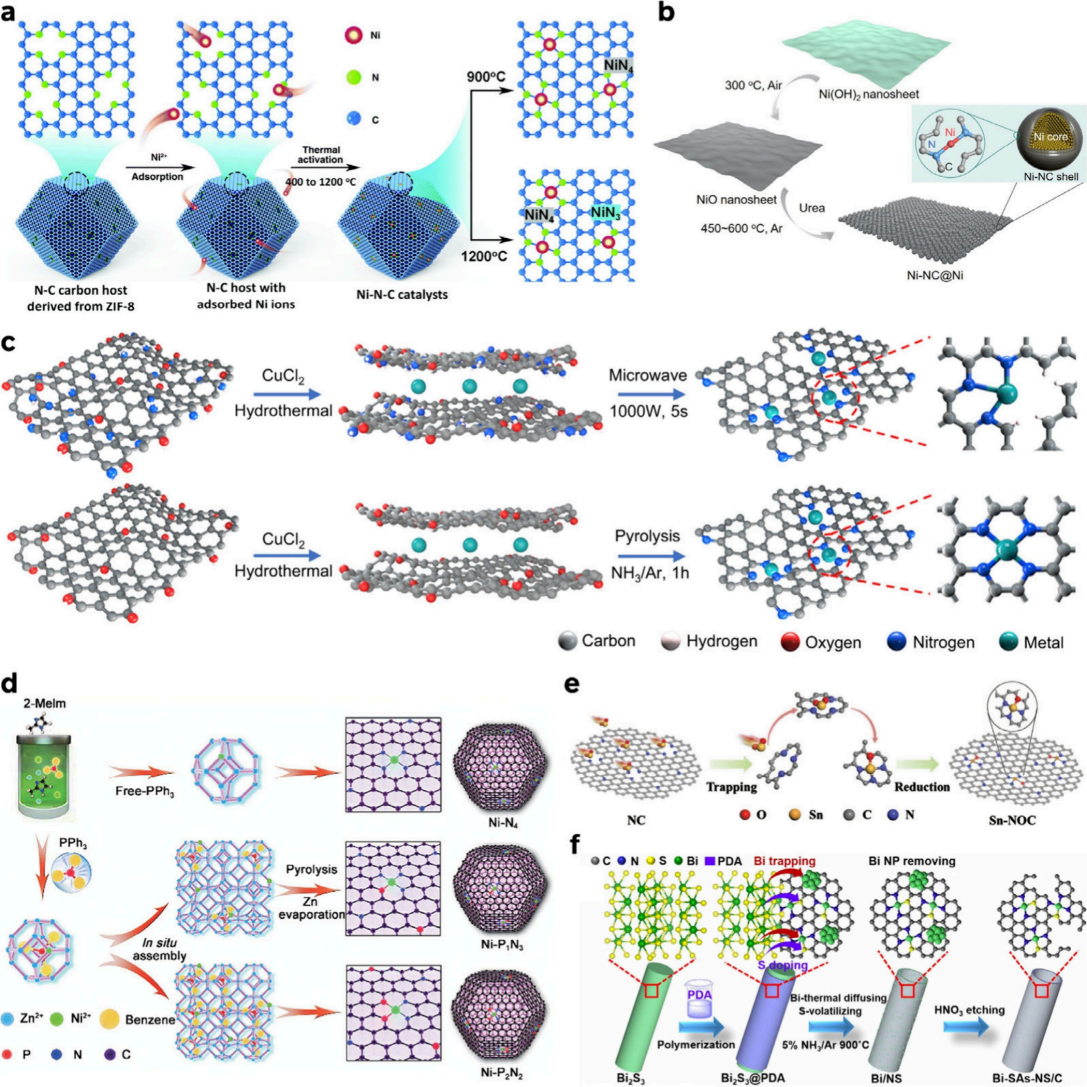
(a) Synthesis scheme of the Ni–N–C catalysts by using a N-doped carbon host to absorb Ni ions followed by thermal activation at different temperatures to tune the Ni–N bond structures and establish structure–property correlations. Reproduced from ref 131. Copyright 2022, Royal Society of Chemistry. (b) Schematic illustration for the formation of the Ni-NC@Ni catalyst. In the green circle: the possible atomic structure of the Ni–N species. Reproduced from ref 132. Copyright 2020, Elsevier. (c) Schematic illustration of the preparation strategy for PSB-CuN_3_ and PS-CuN_4_. Reproduced from ref 133. Copyright 2023, Springer Nature. (d) Schematic illustration for the preparation of Ni–P_*x*_N_*y*_. Reproduced from ref 91. Copyright 2023, Springer Nature. (e) The formation process of atomically dispersed SnN_3_O_1_ active sites. Reproduced from ref 107. Copyright 2021, Wiley-VCH. (f) Schematic illustration of the proposed formation mechanisms. Reproduced from ref 118. Copyright 2021, Springer Nature.

In addition to the aforementioned strategies for synthesizing asymmetric SASCs solely via pyrolysis, the introduction of heteroatoms is another common strategy. For example, Qu et al. successfully constructed SASCs with Ni–N_4_, Ni–P_1_N_3_, and Ni–P_2_N_2_ coordination by simply regulating the content of triphenylphosphine (PPh_3_) confined within the MOF (Figure 1d).^91^ Guo et al. utilized a vapor transport strategy, where gaseous SnO_2_ was trapped by N-doped carbon supports at high temperatures to fabricate Sn SASCs. EXAFS analysis quantitatively determined the first coordination shell of Sn was coordinated with N_3_O_1_, with no detectable Sn–Sn bonds, thereby definitively proving the successful synthesis Sn-NOC with Sn–N_3_O_1_ coordination (Figure 1e).^107^ In a different approach, Wang et al. adopted a simultaneous cation–anion diffusion strategy to synthesize Bi_1_–N_3_S_1_ SASCs. In detail, they used Bi_2_S_3_ as the Bi source, S source, and sacrificial template. Next, under high-temperature pyrolysis conditions (5% NH_3_/Ar, 900 °C), Bi and S species can diffuse into the polydopamine (PDA)-derived N-doped carbon coating on the Bi_2_S_3_ surface. Followed by HNO_3_ etching, Bi-SAs-NS/C SASCs with a Bi_1_–N_3_S_1_ coordination was successfully obtained (Figure 1f).^118^

In summary, the current focus on M_1_-N_*x*_ SASCs research mainly lies in engineering coordination atoms. Nevertheless, the role of the carbon matrix, specifically its defect density, graphitization degree, and heteroatom distribution, is also critical for the stability and electronic structure of the single-atom sites. Defects such as vacancies and edges act as vital anchoring points for immobilizing metal atoms, while the graphitization level dictates the support’s conductivity and thus charge transfer efficiency in ECR.^134,135^ Moreover, the distribution and local concentration of heteroatoms within the carbon framework fine-tune the electron density and ligand field around the metal center, directly modulating the catalytic behavior. Therefore, these aspects deserve further in-depth exploration.^136^ To date, M_1_-N_*x*_ SASCs have emerged as one of the most widely studied electrocatalysts in the field of ECR, largely because they can be synthesized on a gram-scale via simple pyrolysis strategies.^129,137^ Also, some special synthesis methods have been developed, such as electroreduction^138^ and recrystallization.^139^ However, the structure–activity relationship between coordination structures and the CO_2_ catalytic activity remains to be elucidated.

### 2.2 M_1_-M’_*x*_

M_1_-M’_*x*_, also known as single-atom alloys (SAAs), denotes a structure where a target single metal atom (M_1_) is anchored onto a different metal substrate (M’).^140^ Generally, the synthesis of SAAs relies on two principles: (1) the bond energy between the target atom and the host metal is stronger than the bond energy between the host atoms themselves; and (2) the target atoms are present at a very low surface concentration (usually <1%) and are isolated by the host metal atoms.^141^ Unlike traditional disordered alloys, SAAs not only effectively prevent the migration and aggregation of the target single atoms, but also allow the electronic structure of the active sites to be tuned through electronic interactions between the different metals.^79^

Currently, various strategies have been developed to synthesize SAAs for ECR. These mainly include wet-chemistry,^77,79^ galvanic replacement,^142,143^ electroreduction,^78,144^ and pyrolysis.^68,145^ Following this overview, we describe each method individually. As shown in Figure 2a, Liu et al. employed a wet-chemistry method to mix tin (Sn) and copper (Cu) precursors in an alkaline solution, followed by reduction with NaBH_4_. After washing and vacuum drying, they obtained Sn_1_Cu-SAA nanobelts with a length of 2–3 μm, in which isolated Sn atoms were embedded in the Cu lattice.^77^ In another study, Liu et al. prepared Bi nanoparticles via a hydrothermal method and then used controlled addition of RuCl_3_ to replace Bi atoms by galvanic replacement method. This achieved uniform dispersion and stabilization of Ru single atoms (0.6 wt %) on the Bi support, constructing a Ru_1_@Bi SAA catalyst (Figure 2b).^143^ Jin et al. successfully synthesized a novel Mo_1_Cu SAA through a stepwise chemical conversion combined with an *in situ* electroreduction strategy, which is at ambient temperature in a typical flow cell (Figure 2c).^144^ Moreover, Tuo et al. adopted a stepwise pyrolysis approach in which they synthesized a NiZn bimetallic metal organic framework (MOF) precursor via a hydrothermal method and then performed controlled pyrolysis at different temperatures (700, 800, and 900 °C). The volatility of Zn at high temperatures was utilized to adjust its residual content, successfully constructing carbon-supported NiZn bimetallic catalysts. Among them, the NiZn_0.03_/C sample obtained after pyrolysis at 900 °C possessed a strongly electronically coupled Ni–Zn dual-site structure. This led to a moderate downshift of the *d*-band center, achieving the Sabatier-optimal adsorption balance during the process of ECR (Figure 2d).^145^

**Figure 2:**
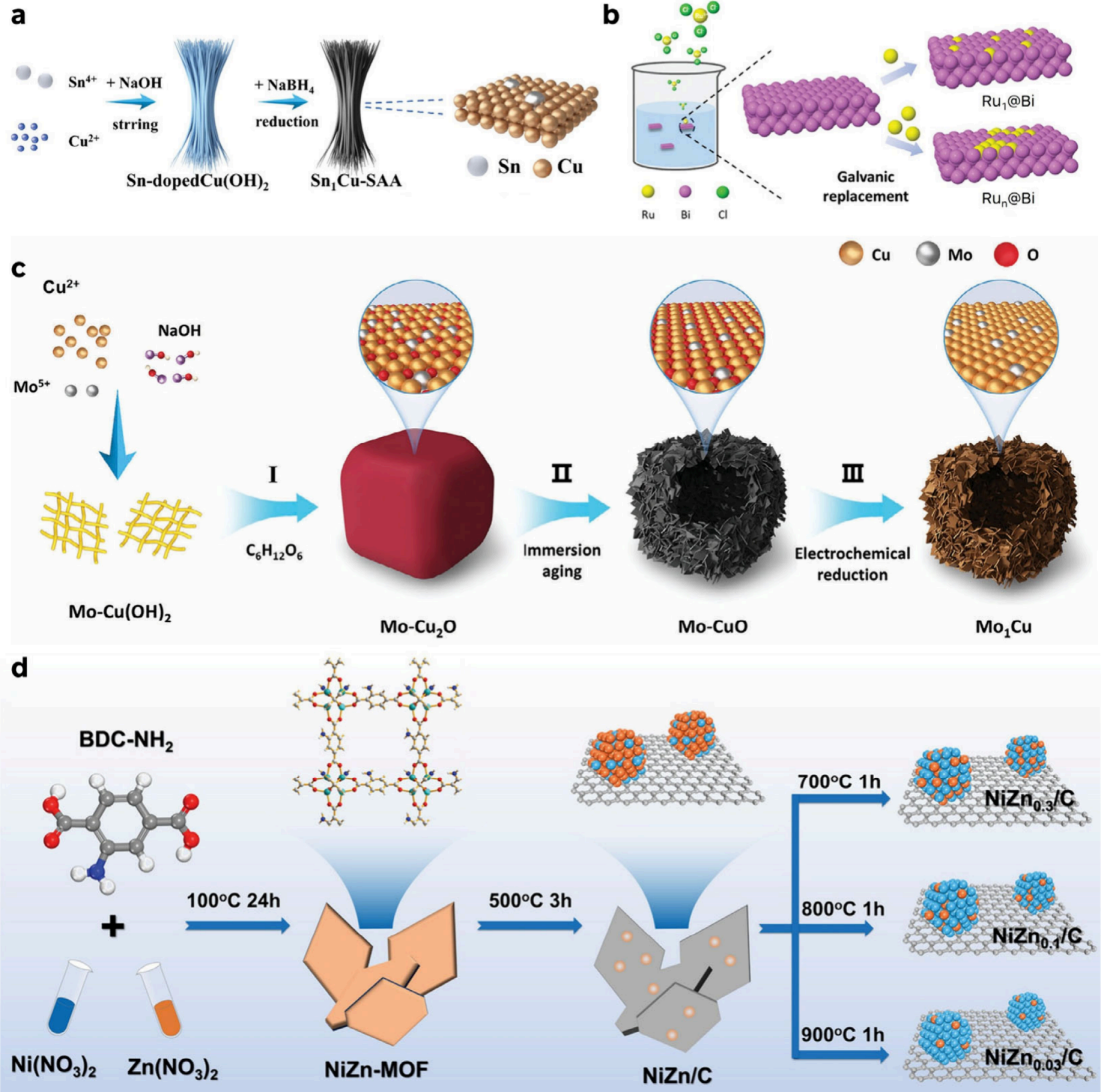
(a) Schematic synthesis process of the Sn_1_Cu-SAA. Reproduced from ref 77. Copyright 2025, Wiley-VCH. (b) Schematic representation of the preparation of the Ru_1_@Bi and Ru_n_@Bi catalysts. Reproduced from ref 143. Copyright 2024, Wiley-VCH. (c) Schematic diagram for the preparation of Mo_1_Cu catalysts. Reproduced from ref 144. Copyright 2025, Wiley-VCH. (d) Schematic diagram of the NiZn_0.03_/C bimetallic catalyst synthesis process. Reproduced from ref 145. Copyright 2023, Wiley-VCH.

Apart from the synthesis methods introduced above, photodeposition,^146^ laser ablation,^147^ and physical vapor deposition (PVD)^148^ are also used to prepare single-atom alloy catalysts. However, these methods are rarely applied in the field of ECR, possibly due to the limited accessibility of the required specialized equipment, thus presenting research opportunities for further exploration.

### 2.3 M_1_-O_*x*_

Similar to the definitions of M_1_-N_*x*_ and M_1_-M’_*x*_, M_1_-O_*x*_ is defined as an active site where a single metal atom is anchored by multiple electronegative O atoms (usually ≤4).^149^ However, M_1_-O_*x*_ are mainly supported on metal oxides,^83,150–153^ MOFs,^154,155^ and MXenes,^156^ rather than carbon materials.^7^ This sharp contrast to M_1_-N_*x*_ can be attributed to the specific bonding nature of O atoms, which, with six valence electrons and a tendency to form two bonds, typically exist as unstable surface or edge functional group on carbon supports instead of lattice integration.^157,158^ In contrast, the O atoms in metal oxides, MOFs, and MXenes are part of the rigid skeleton, allowing them to firmly anchor single metal atoms and form stable M_1_-O_*x*_ structures.^159^

In recent years, metal oxide-supported M_1_-O_*x*_ SASCs have attracted significant attention in the ECR field due to their industrial importance. Their synthesis strategies mainly include impregnation,^159,160^ Li-molten salt,^158,161^ and coprecipitation.^162^ Among them, impregnation can be simply described as introducing metal precursors onto a prepared support and converting them into single-atom sites during subsequent treatment.^52^ For example, Guo et al. synthesized pure monoclinic BiVO_4_ via a solvothermal method. Then, they removed Bi by using NaBH_4_ reduction to create Bi vacancies. Finally, Cu single atoms were stably anchored onto these vacancies through electrostatic interactions, successfully producing Bi_1–*x*_VO_4_–Cu SASCs with a Cu loading of ∼ 1 wt % (Figure 3a).^160^ An alternative synthetic strategy is Li-molten salt, which leverages the low melting point of lithium (180 °C) and its capability to disperse metals. Based on this, SASCs are prepared by achieving atomic-level dispersion through methods like ultrasonication.^163^ For instance, Xu et al. successfully synthesized Cu–C catalyst coordinated with O from hydroxyl and carboxyl groups by mixing Cu bulk with molten lithium, followed by steps including ultrasonic dispersion, quenching, air humidification, and water washing (Figure 3b).^158^ For the coprecipitation strategy, it can also be used to synthesize SASCs. In this method, two or more metal cations in a solution react with a precipitant, followed by air calcination to obtain SASCs. For example, Yang et al. used the reducing effect of NH_3_·H_2_O to coprecipitate Cu­(NO_3_)_2_·3H_2_O and ZrO­(NO_3_)_2_·xH_2_O into a Co-p-CuZr-0.0 precursor. After calcining at 500 and 900 °C for 3 h, they successfully prepared Cu single-atom catalysts supported on ZrO_2_ with two different crystal phases, namely Cu_1_O_3_-tZrO_2_ and Cu_1_O_4_-mZrO_2_ (Figure 3c).^83^

**Figure 3:**
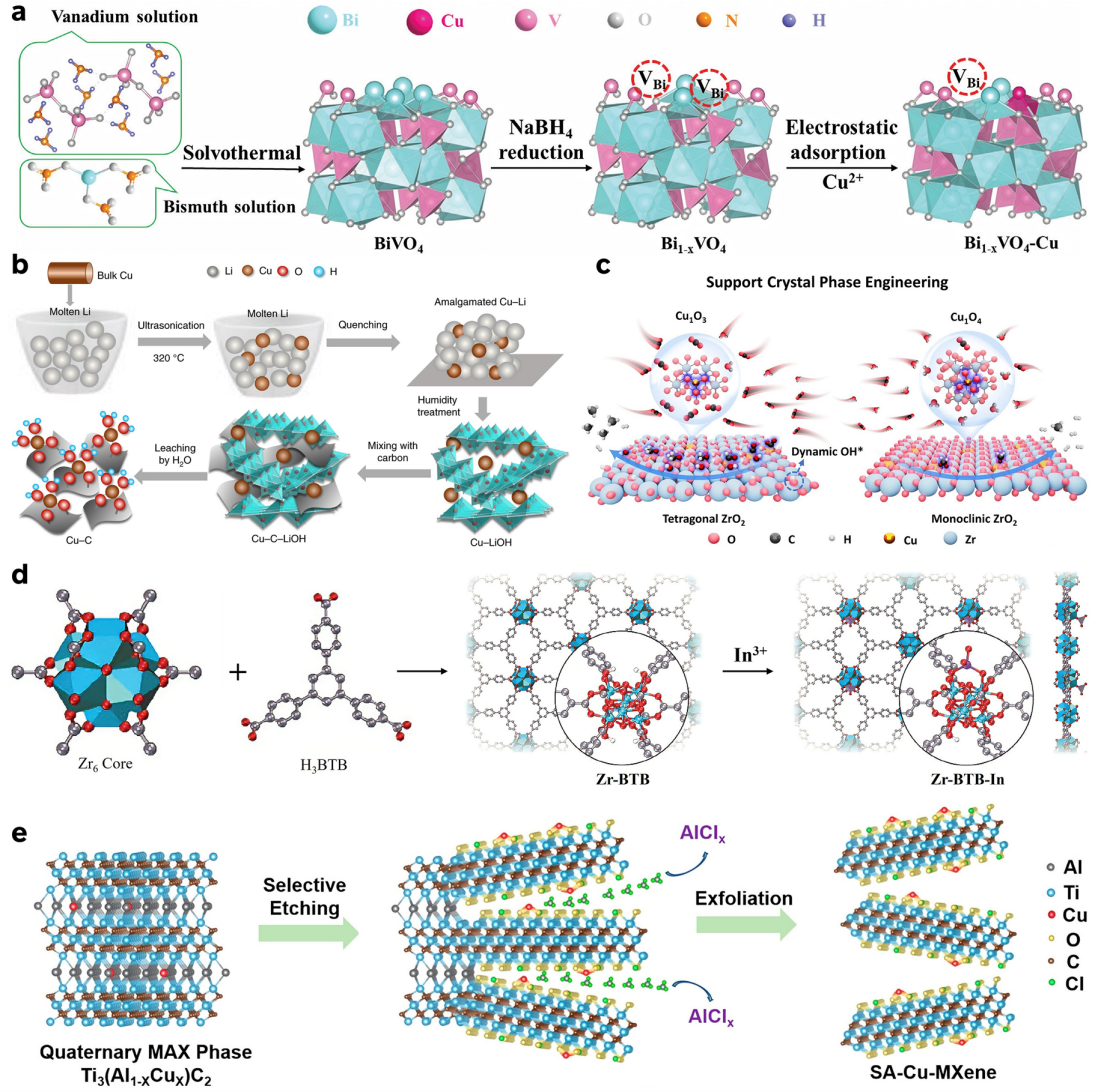
(a) Schematic illustration of preparing Bi_1–*x*_VO_4_–Cu. Reproduced from ref 160. Copyright 2024, Wiley-VCH. (b) Step-by-step preparation of the carbon-supported Cu SA catalyst using an amalgamated Cu–Li method. Reproduced from ref 158. Copyright 2020, Springer Nature. (c) Schematic illustration of the support crystal phase engineering and reaction mechanisms on the surface of Cu_1_O_3_-tZrO_2_ and Cu_1_O_4_-mZrO_2_. Reproduced from ref 83. Copyright 2025, American Chemical Society. (d) Illustration of the synthesis of Zr-BTB-In. Reproduced from ref 154. Copyright 2025, Wiley-VCH. (e) Schematic illustration of the fabrication of SA-Cu-MXene via selective etching of quaternary MAX-Ti_3_(Al_1–*x*_Cu_*x*_)­C_2_. Gray, blue, red, yellow, brown, and green balls represent Al, Ti, Cu, O, C, and Cl atoms, respectively. Reproduced from ref 156. Copyright 2021, American Chemical Society.

In addition to metal oxides, MOFs and MXenes can also serve as supports for the M_1_-O_*x*_ SASCs. For example, Li et al. synthesized an ultrathin 2D MOF (Zr-BTB) using ZrCl_4_ and 1,3,5-Tris (4-carboxyphenyl) benzene (BTB) via a solvothermal method. Then, they stirred Zr-BTB in InCl_3_ at 60 °C for 12 h to synthesize Zr-BTB-In catalyst with an In loading of about 2.5 wt % (Figure 3d).^154^ Zhao et al. strategically introduced ZnCl_2_ during the synthesis of the SA-Cu-Mxene catalyst, where it reacts with aluminum (Al) during pyrolysis (600 °C, 5 °C/min, 5 h) to form volatile AlCl_3_. This allows unreacted Cu to remain loaded on the MXene skeleton. Finally, the SA-Cu-Mxene catalyst with Cu–O_3_ coordination was successfully synthesized (Figure 3e).^156^

Overall, compared to M_1_-N_*x*_ SASCs mentioned in the previous sections, there are fewer studies on M_1_-O_*x*_ SASCs in the ECR field, which can be attributed to the higher synthetic complexity relative to the simple pyrolysis strategy. In addition, synthesis strategies such as ball milling,^164^ atomic layer deposition (ALD),^165^ and microemulsion^52^ are rarely seen in this area. However, M_1_-O_*x*_ SASCs still represent a promising area for further exploration in ECR research.

## 3 Coordination Detection of SASCs

The previous section systematically reviewed the precise synthesis strategies for three main coordination types (M_1_-N_*x*_, M_1_-M’_*x*_, and M_1_-O_*x*_) of SASCs, providing practical pathways for their preparation and laying a foundation for investigating their structure–activity relationships in the field of ECR. In particular, it is worth noting that the fine coordination structures of SASCs currently rely on advanced characterization techniques such as AC-HAADF-STEM, XAFS (e.g., X-ray absorption near edge structure (XANES) and EXAFS), and Mössbauer spectroscopy. Nevertheless, these techniques still have intrinsic limits. Therefore, building upon a detailed review of the historical evolution of each technique, this chapter also critically evaluates their respective strengths and limitations to provide reference for future research.

### 3.1 AC-HAADF-STEM

AC-HAADF-STEM has undergone nearly a century of development. In the 1930s, Manfred von Ardenne attempted to replace visible light with electron beams of extremely short wavelengths, developing the world’s first scanning transmission electron microscope (STEM).^166,167^ However, its resolution remained severely limited for decades due to the low brightness of thermal cathodes and the inherent spherical aberration (pointed by Scherzer^168^ in 1936) of electromagnetic lenses. A turning point arrived in 1970 when Crewe introduced a high-brightness field emission gun (FEG) combined with an annular dark-field (ADF) detector, successfully capturing blurry images of uranium (U) and thorium (Th) atoms on a carbon film using STEM for the first time.^169^ Subsequently, in the 1990s, Pennycook refined the high angle ADF (HAADF) imaging theory, clarifying that collecting high-angle incoherent scattered electrons enables ″atomic number (Z)-contrast″ imaging. In this mode, intensity is proportional to Z^2^, thereby offering a robust basis for directly distinguishing heavy atoms.^170^

Nevertheless, image clarity remained limited by spherical aberration of lens defects until the late 1990s, when Haider and Krivanek successfully developed the spherical aberration corrector. Acting like ″corrective glasses″ for the microscope, this device historically compressed the electron beam probe to the subangstrom scale (<0.1 nm).^173,174^ Thus, the STEM with a perfect combination of aberration correction (AC), the HAADF, and other components (Figure 4a) offers extremely high resolution and exceptional identification of heavy atoms. Following the pioneering work of Zhang et al. in 2011, which achieved the direct observation of Pt single atoms on FeO_*x*_ (Figure 4b), this technique was established as the ″gold standard″ for characterizing atomic-level microstructures, particularly for visualizing the dispersion of single atoms.^44^

**Figure 4:**
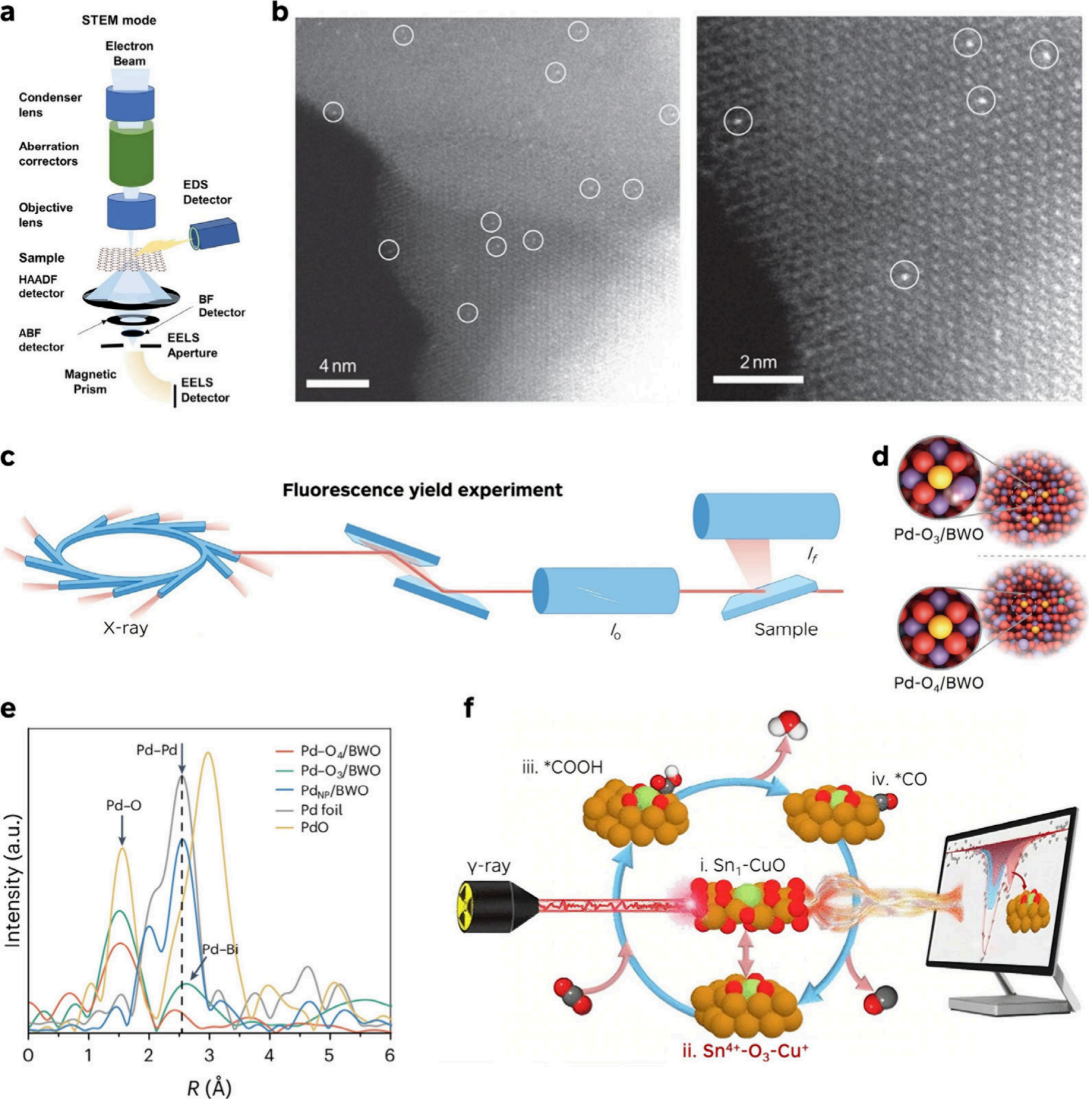
(a) General schematic of an electron microscope in STEM modes with a condenser lens, objective lens, aberration correctors, projector system, retractable detectors, EDS (energy dispersive X-ray spectroscopy), and EELS (electron energy loss spectroscopy) acquisition. p.s. BF (bright-field), ABF (annular bright-field). Reproduced from ref 171. Copyright 2021, Wiley-VCH. (b) Pt single atoms (white circles) are seen to be uniformly dispersed on the iron oxide (FeO_*x*_) support (left) and occupy exactly the positions of the Fe atoms (right). Reproduced from ref 44. Copyright 2011, Springer Nature. (c) Schematic diagram of XAFS measurements with fluorescence yield experiment setup. Reproduced from ref 74. Copyright 2024, Springer Nature. (d) Schematic diagram of Pd–O_4_/BWO and Pd–O_3_/BWO. (e) Fourier transforms of the EXAFS spectra of the Pd K-edge spectra. Reproduced from ref 172. Copyright 2025, Springer Nature. (f) Schematic diagram of *operando* Mössbauer spectroscopy for tracking the metastable states of atomically dispersed tin (Sn) in copper oxide (CuO) for selective CO_2_ electroreduction. Reproduced from ref 162. Copyright 2023, American Chemical Society.

### 3.2 XAFS

The evolution of XAFS technology dates back to 1920, when Fricke first observed oscillatory phenomena on the high-energy side (>200 eV) of the K-edge absorption spectra for elements ranging from magnesium (Mg) to chromium (Cr), though the underlying mechanism remained unexplained.^175^ In 1931, Kronig attempted to clarify it by proposing the Long-Range Order (LRO) theory.^176^ However, he erroneously attributed the oscillations to Bragg diffraction of photoelectrons within the lattice’s periodic potential. This led to a widespread misconception that materials lacking long-range order were incapable of generating XAFS signals, thereby severely impeding the technique’s application.

A paradigm shift was initiated in 1971 by the seminal publication of Sayers, Stern, and Lytle (SSL).^177^ They confirmed that oscillatory phenomena on the high-energy side essentially originate from the quantum interference effect between the outgoing photoelectron wave and the backscattered wave from neighboring atoms by proposing Short-Range Order (SRO) theory. Also, they introduced the use of Fourier Transform to convert energy-space (*k*-space) oscillations into a radial distribution function (*R*-space), enabling the direct extraction of local structural parameters, including coordination types, numbers and bond lengths. This breakthrough marked the inception of modern XAFS, establishing it as a potent tool for investigating noncrystalline materials. Concurrently, the invention of high-brightness synchrotron radiation sources (10^4^ to 10^6^ times brighter than traditional X-ray tubes) greatly improved data acquisition efficiency and signal-to-noise ratios, paving the way for *in situ* investigations.^178^ On the theoretical front, the multiple scattering theory developed by Rehr and Albers in the 1990s further advanced the field, allowing for the construction of atomic oxidation states and 3D geometries via XANES.^179^

In the 21st century, with the subangstrom resolution in analyzing coordination environments (e.g., coordination types and numbers) of XAFS, SASCs have entered a golden age for identifying single-atom active sites (such as M–N-C). Given the relatively low metal loading of SASCs (typically <1 wt %), XAFS measurements are predominantly conducted in fluorescence mode rather than transmission mode using a multielement solid-state detector (e.g., Ge or Si drift detector) to ensure a sufficient signal-to-noise ratio (Figure 4c).^74,180^ For example, Li et al. used EXAFS technique to confirm the Pd–O_4_ and Pd–O_3_ coordination structure in the synthesized Pd–O_4_/BWO and Pd–O_3_/BWO catalysts with a Pd loading of approximately 0.05 wt % (Figure 4d, e).^172^

### 3.3 Mössbauer Spectroscopy

In contrast to XAFS that relies on the photoelectric effect excited by X-rays, Mössbauer spectroscopy mainly operates on a unique principle utilizing high-energy γ-rays, which is named as ″recoil-free nuclear resonant fluorescence″ observed by Rudolf Mössbauer in 1958.^181^ However, this initial observation required embedding ^191^Iridium (Ir) atoms in a solid lattice at cryogenic temperatures, suffering from harsh experimental requirements and limited versatility. A major breakthrough occurred with the in-depth investigations by Pound et al. on the Mössbauer effect of ^57^Fe. Due to its low γ-ray energy (14.4 keV) and ubiquity in nature and industry, ^57^Fe propelled this technology into an era of rapid development.^182,183^

While XAFS provides coordination structures (e.g., coordination types and numbers) of SASCs, Mössbauer spectroscopy offers site-average resolution sensitive to subtle electronic variations, enabling the precise identification of active sites with identical coordination but different geometries or spins.^184^ For instance, Li et al. utilized this technique to deconvolute Fe–N_4_ sites into two distinct types: D1 (doublet with QS = 0.9–1.2 mm·s^–1^) and D2 (doublet with QS = 1.8–2.8·s^–1^).^185^ Chen et al. tracked the structural evolution of single Tin (Sn) atoms on a copper oxide (CuO) support under ECR conditions via *operando* Mössbauer spectroscopy. This finding clearly demonstrated a transformation from Sn^4+^-O_4_–Cu^2+^ to a metastable Sn^4+^-O_3_–Cu^+^ configuration, providing critical insights into the role of single Sn atoms in tuning the electronic structure of the catalyst (Figure 4f).^162^

### 3.4 Intrinsic Limits

Although AC-HAADF-STEM, XAFS, and Mössbauer spectroscopy collectively constitute the cornerstone for characterizing the coordination environments of SASCs, it must be acknowledged that no single technique offers a flawless resolution of all structural details. In the following section, the intrinsic limits of each advanced characterization technique are critically discussed.

AC-HAADF-STEM, despite its capability to achieve atomic-level resolution, is severely limited by electron irradiation damage (e.g., knock-on damage and ionization damage) arising from high-energy electron beams (accelerating voltage up to 300 kV).^189^ For M_1_-N_*x*_ and M_1_-M’_*x*_ SASCs, the high density of delocalized electrons can facilitate the ultrafast quenching (<1 fs) of holes generated by incident electrons. Consequently, the typically more destructive ionization damage is effectively suppressed, leaving knock-on damage, where incident electrons elastically displace atoms via momentum transfer, as the dominant mechanism (Figure 5a).^186^ To address this, lowering the accelerating voltage (30–80 kV) is a simple but useful strategy to preserve the pristine structure of SASCs.^190^ Conversely, for generally poorly conductive M_1_-O_*x*_ SASCs, ionization damage induced by inelastic scattering usually predominates, as holes generated by incident electrons cannot be rapidly neutralized, resulting in lifetimes often exceeding the period of atomic vibration. During this interval, the electronic wave functions can be perturbed (e.g., via vibrational excitation), and a portion of the excitation energy is stored as potential energy, leading to bond destabilization and structural degradation (Figure 5b).^187^ Herein, reducing the electron dose or increasing the accelerating voltage energy (to decrease the differential scattering cross-section) are effective to minimize the destruction of SASCs active sites.^191^ Also, it is crucial to note that AC-HAADF-STEM only sees a very small area (typically nanometers), which makes it hard to represent the whole sample. In other words, we cannot confirm whether the metal species of SASCs are uniformly dispersed as single atoms based on just a few tiny images. Therefore, it is necessary to combine it with other advanced characterization techniques such as XAFS.

**Figure 5:**
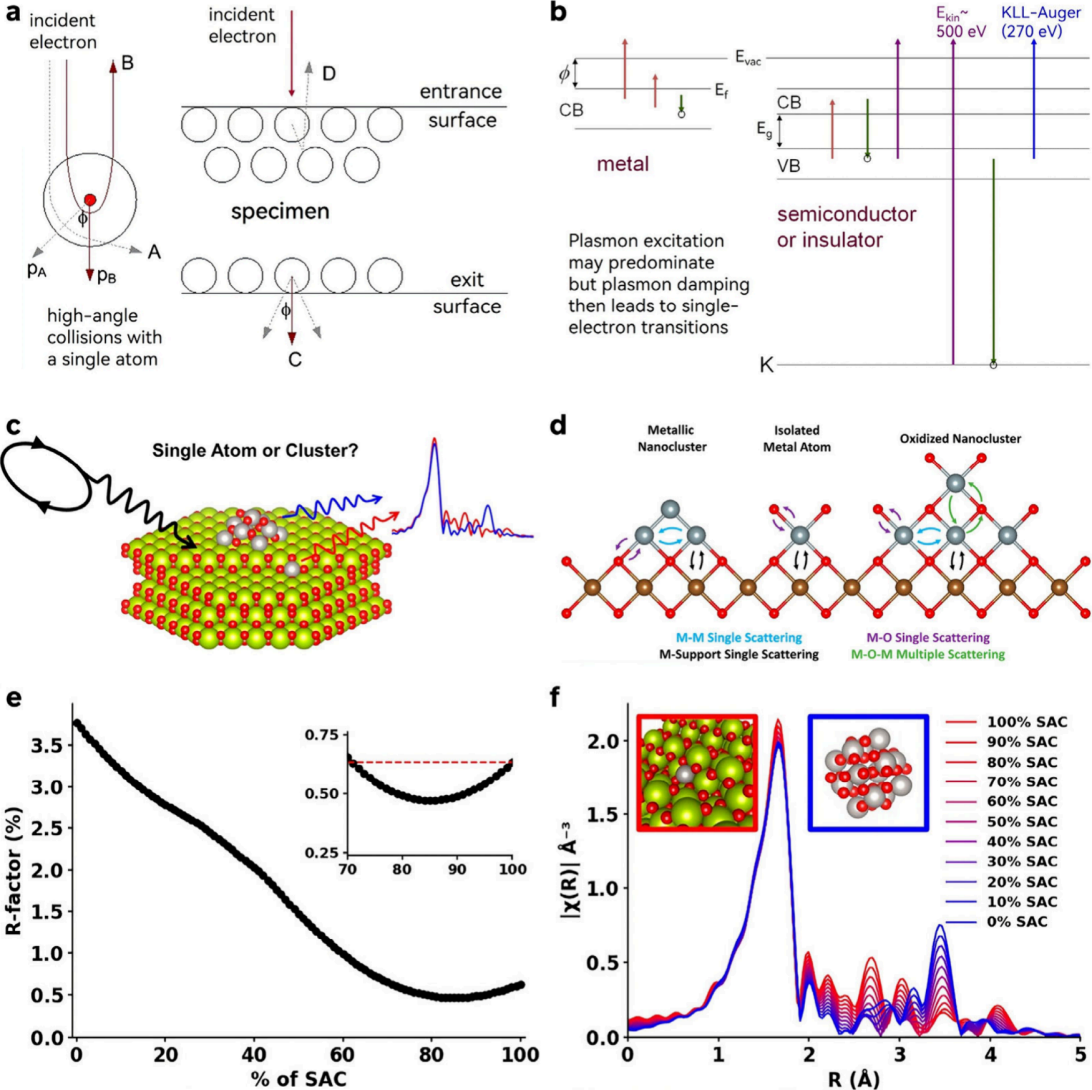
(a) Elastic scattering of electrons from an atomic nucleus, shown schematically (particle model) for a large-angle collision (A) and a 180° collision (B). Sputtering of atoms from the beam-exit surface (C) and the beam-entry surface (D). Reproduced from ref 186. Copyright 2010, Elsevier. (b) Energy-band diagram of a metal (left) and a semiconductor or insulator (right), electron energy being plotted vertically upward. CB and VB represent the conduction and valence bands, f is the work function, E_vac_, E_f_ and E_g_ are the vacuum energy, Fermi energy, and CB-VB energy gap. Upward arrows represent single-electron excitations, while downward arrows are de-excitation processes (filling of a VB or K-shell hole). K-shell excitation in an organic material is shown on the right, with E_kin_ being the typical kinetic energy of an excited K-shell electron. This process also results in the emission of Auger electrons from the VB, with an energy of about 270 eV. Reproduced from ref 187. Copyright 2013, Elsevier. (c) Schematic diagram of heterogeneous Pt/CeO_2_ SASCs using EXAFS. (d) Scattering paths for SASCs and small clusters. Schematic depiction of scattering paths present in oxide-supported metal catalysts with a heterogeneity of the metal-site structure including isolated metal sites (middle) as well as metal (left) or oxidized (right) nanoclusters. Bonds are for representation only. Color code: metal (gray), oxygen (red), and support metal cation (brown). (e) R-factor of the 5-path SAC fit to data sets with varying contributions from the Pt/CeO_2_ SASCs site and metallic Pt_13_ cluster. The inset shows a zoomed-in region with 70–100% SAC sites (30–0% Pt_13_ cluster sites). Data are taken over Δk = 3–18 Å^–1^. (f) Modeled FT EXAFS data for mixtures of the Pt/CeO_2_ SASCs site and metallic Pt_13_ cluster. Reproduced from ref 188. Copyright 2023, American Chemical Society.

Currently, XAFS analysis is a powerful tool for characterizing the coordination environment of SASCs. Specifically, the XANES provides insights into the metal oxidation state, electronic properties, and bonding geometry, while the EXAFS reveals the local coordination environment of atom type, number, and bond length (within ∼5 Å).^192^ Therefore, EXAFS is widely employed to detect the absence of metal clusters in SASCs. However, since its signals are dominated by the scattering paths of the major species, the resulting spectrum represents a bulk averaged structure if the sample contains mixed species (e.g., single atoms and nanoclusters), potentially masking the true configuration of the active sites.^193,194^ Moreover, the EXAFS peak intensity may also sharply decrease with small particle size.^195,196^ For instance, Finzel et al. demonstrated that the R-factor (a quality of fit metric that quantifies the misfit between data and model) of EXAFS lacks sensitivity to the scattering paths (e.g., M-M, M-O, and M-O-M) of small mixed species in Pt/CeO_2_ (Figure 5c, d). In detail, even when the Pt/CeO_2_ catalyst contained approximately 40% Pt_14_O_37_ clusters (d: ∼1 nm), the R-factor derived from a pure single-atom model remained below 1%, satisfying the conventional standard for a ″good″ fit (Figure 5e, f). Additionally, this insensitivity can be visually confirmed by the barely discernible changes in the simulated Fourier transformation (FT)-EXAFS data.^188^ These findings imply that relying solely on a low R-factor is statistically insufficient to confirm the absence of small clusters in SASCs. Therefore, it is recommended to carefully scrutinize the features of scattering paths (e.g., M-M, M-O, and M-O-M) in the high R-space, employ linear combination fitting to quantitatively assess the potential presence of clusters, and incorporate continuous Cauchy wavelet transform (CCWT) to assist in identifying weak cluster signals.^197,198^

For Mössbauer spectroscopy, despite its exquisite sensitivity to electronic structures of SASCs, it also suffers from a critical inherent limitation. Although Mössbauer effect has been observed in approximately 40 elements, suitable Mössbauer-active isotopes (e.g., ^57^Fe, ^119^Sn, ^121^Sb, and ^151^Eu) remain rare when considering the technical and analytical challenges.^199^ Herein, this technique is frequently employed as a specialized complementary technique in conjunction with AC-HAADF-STEM and XAFS to provide unique insights into oxidation states, spin states and coordination symmetries of SASCs.^180^

In summary, to obtain comprehensive and accurate coordination structures of SASCs, researchers must adopt a ″multitechnique combination″ strategy, leveraging the complementary strengths of AC-HAADF-STEM, XAFS, and Mössbauer spectroscopy to cross-validate findings and overcome the inherent bottlenecks of individual characterization techniques.

## 4 Structure–Activity Relationship of SASCs

The preceding sections have comprehensively elaborated on the precise synthesis strategies and structural characterization of SASCs with diverse coordination types, establishing a solid material foundation for an atomic-level understanding of the structure–activity relationships in the field of ECR. Then, it is essential to recognize that ECR is intrinsically a complex PCET process (Figure 6). Table 1 presents the standard potentials (E^0^, vs SHE) for ECR and the competitive HER in aqueous media. Notably, an external driving force is necessary to overcome the barrier for the initial reduction of the mass of CO_2_. Following this, diverse hydrocarbons are generated via PCET process.^200^ As the number of electron transfers increases (2e^–^ to 18e^–^), the reaction pathways exhibit high diversity, resulting in a broad product distribution from C_1_ products (e.g., 2e^–^ CO and HCOOH, up to 8e^–^ CH_4_) to complex C_2+_ products (e.g., 10e^–^ CH_3_CHO, 12e^–^ C_2_H_4_ and C_2_H_5_OH, and even 18e^–^ CH_3_CH_2_CH_2_OH).^39^ Although the specific reaction steps of ECR may initially appear intricate, a broad consensus based on the Sabatier principle and density functional theory (DFT) calculations has suggested that the adsorption energy of key intermediates on the highly uniform active centers is the governing factor determining catalytic performance.^201^ In light of this, this section discusses the rational design principles of SASCs tuning for specific target products, incorporating current insights into the ECR mechanism.

**Figure 6:**
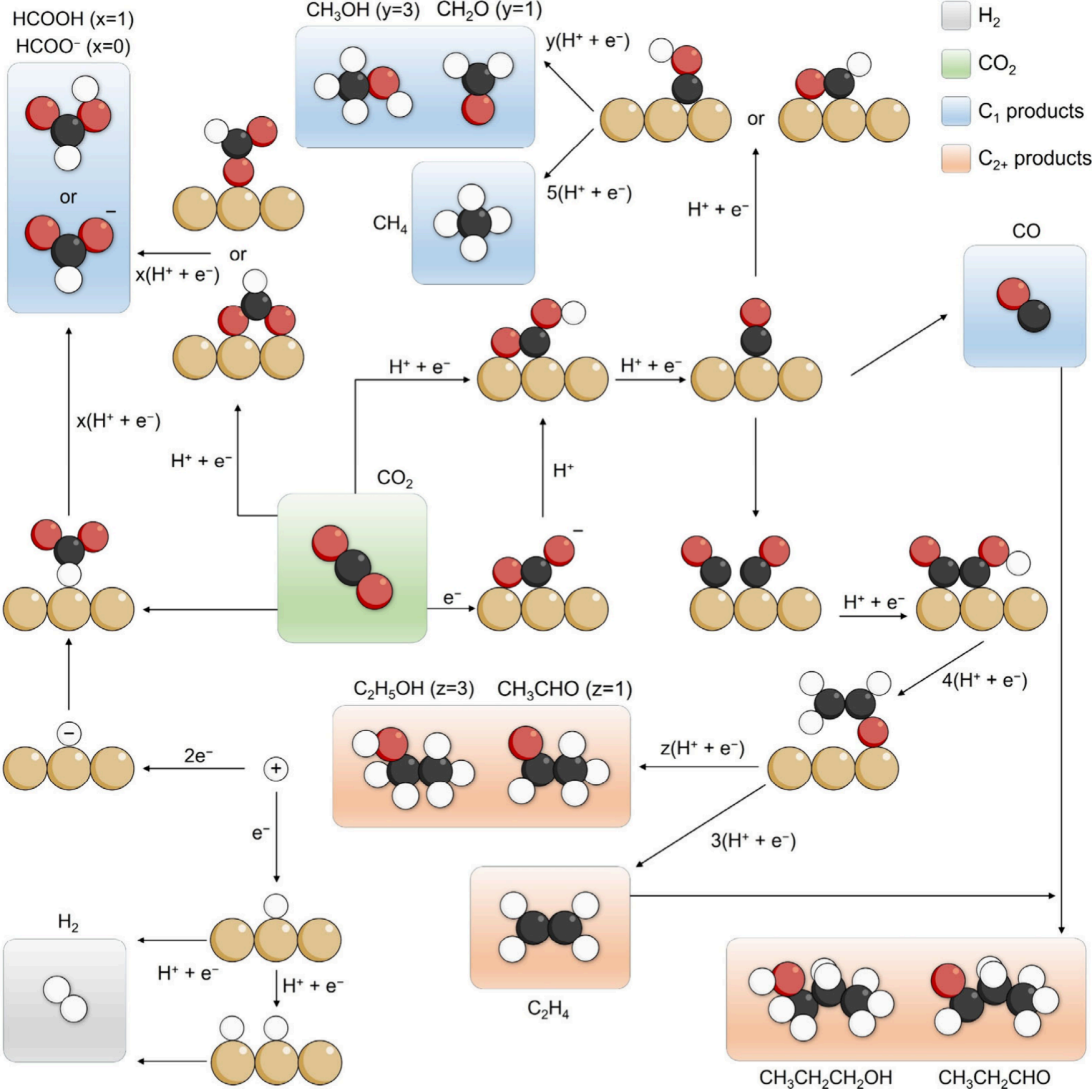
Overview of reaction pathways for ECR toward different products. Black spheres, carbon; red spheres, oxygen; white spheres, hydrogen; blue spheres, (metal) catalyst. The arrows indicate whether proton, electron or PCETs take place. Reproduced from ref 39. Copyright 2019, Springer Nature.

**Table 1:** Standard Potentials of ECR in Aqueous Media at 1 atm and 25 °C

Half-cell reaction	Standard potentials(V vs SHE)
2H^+^ + 2e^–^ → H_2_	–0.42
CO_2_ + e^–^ → *CO_2_^–^	–1.9
CO_2_ + 2H^+^ + 2e^–^ → CO + H_2_O	–0.52
CO_2_ + 2H^+^ + 2e^–^ → HCOOH	–0.61
CO_2_ + 4H^+^ + 4e^–^ → HCHO + H_2_O	–0.51
CO_2_ + 6H^+^ + 6e^–^ → CH_3_OH + H_2_O	–0.38
CO_2_ + 8H^+^ + 8e^–^ → CH_4_ + 2H_2_O	–0.24
2CO_2_ + 12H^+^ + 12e^–^ → C_2_H_4_ + 4H_2_O	–0.34
2CO_2_ + 12H^+^ + 12e^–^ → C_2_H_5_OH + 3H_2_O	–0.33

### 4.1 CO_2_ to C_1_ Products

As illustrated in Figure 6, the major C_1_ products, including CO, HCOOH, CH_2_O, CH_3_OH, and CH_4_, are highlighted in blue boxes. It is generally accepted that *COOH serves as the key intermediate for the conversion of CO_2_ to CO. This pathway can proceed via a PCET process or a sequential electron transfer-proton transfer (ET-PT) route.^200,202,203^ Subsequently, derived from the PCET reduction of *COOH, the *CO species is widely regarded as the pivotal intermediate for the synthesis of CH_2_O, CH_3_OH, and CH_4_.^204^ In contrast, the key intermediate for HCOOH or HCOO^–^ remains controversial, and the proposed mechanisms mainly involve either *OCHO adsorbed via single/double oxygen atoms (monodentate/bidentate) or *HCOO formed via an anionic hydride-mediated pathway.^39,205^ Based on these consensuses, the following section explores the rational design principles of SASCs tailored for specific C_1_ products.

#### 4.1.1 CO

ECR-to-CO represents a promising dual-benefit strategy, as it not only mitigates global warming but also facilitates the carbon-neutral cycle by producing CO, a vital feedstock for the Fischer–Tropsch process and other industrial synthesis.^206^ Recent literature indicates that catalysts capable of highly efficient CO production are predominantly centered on M_1_-N_*x*_ SASCs, with a smaller portion involving M_1_-M’_*x*_ and M_1_-O_*x*_ SASCs. Accordingly, we will discuss these types of SASCs for ECR-to-CO in sequence.

To date, most reported M_1_-N_*x*_ SASCs (particularly those based on transition metals such as Ni,^81^ Fe,^207^ and Co^115^) have exhibited FE_CO_ exceeding 90%. However, it remains challenging to directly compare the intrinsic activities, selectivities, and reaction mechanisms of different metal centers due to the structural heterogeneity inherent in their synthesis. Herein, Chang et al. employed a series of transition-metal porphyrins (TM-Pcs) as well-defined model platforms to systematically investigate the intrinsic roles of isolated transition metals (Fe, Co, Ni, Cu, and Zn) in ECR-to-CO research (Figure 7a). Their findings revealed that Co-Pc exhibited the most superior CO activity (FE = 95%, E = −0.7 V vs RHE, *j*_CO_ = 8.6 mA·cm^–2^). *In-situ* XAFS confirmed that the TM-N_4_ coordination structure remained unchanged during ECR, which can be attributed to the strong covalent interactions between the transition metal (TM) and N atoms. Further DFT calculations elucidated that the moderate binding strength of the Co-Pc active center toward key intermediates (*HOCO and *CO) was the mechanistic origin of its exceptional performance (Figure 7b).^116^ Although Ni-Pc exhibits higher energy barriers for *HOCO and *CO than Co-Pc, leading to lower CO activity, its low-cost and modifiable electronic structure have driven extensive research into Ni-based SASCs for CO production. For instance, Wang et al. introduced the S atom (∼2.58), which is less electronegative than the N atom (∼3.04), to form a Ni–N_3_S coordination structure, thereby increasing the *d*-orbital electron density of the Ni atoms. DFT calculations demonstrated that the Ni–N_3_S site possesses a lower energy barrier for *COOH formation compared to that of the Ni–N_4_ site. Experimentally, the Ni–N_3_S–C SASCs achieved maximum FE_CO_ of 98.6%, 94.8%, and 90.5% at a current density of 100 mA·cm^–2^ in alkaline, acidic, and neutral electrolytes, respectively (Figure 7c).^102^

**Figure 7:**
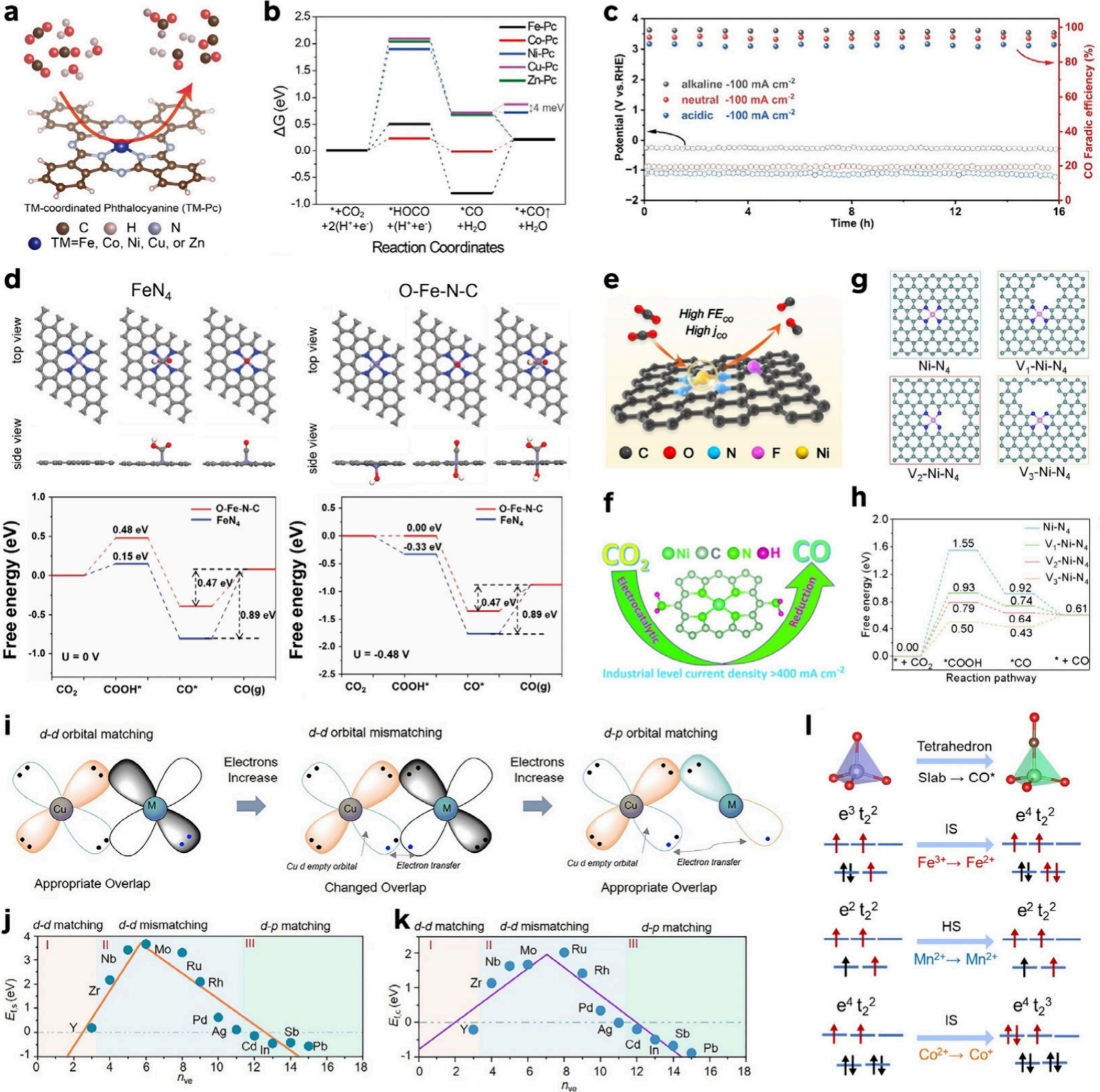
(a) Schematic illustration of CO_2_RR over a chemical unit of the TM-Pcs. (b) Free energy diagram of TM-PCs for CO_2_RR at V = 0 V. Reproduced from ref 116. Copyright 2022, American Chemical Society. (c) Stability test of Ni–N_3_S–C in flow cell with alkaline, acidic, and neutral electrolyte. The electrolytes are 1 M KOH (alkaline, pH = 14), 0.05 M H_2_SO_4_ + 1 M KCl (acidic, pH = 1), and 1 M KHCO_3_ (neutral, pH = 8.2). Reproduced from ref 102. Copyright 2025, Wiley-VCH. (d) Top-left, top-right: Top view and side view optimized adsorption configuration on simulated FeN_4_ and O–Fe–N-C (Fe, O, N, and C atoms are represented in purple, red, blue, and gray, respectively). Down-left, down-right: Free energy profiles for the CO_2_RR to CO at 0 V (vs RHE) and at – 0.48 V (vs RHE) on simulated FeN_4_ and O–Fe–N-C. Reproduced from ref 208. Copyright 2022, Wiley-VCH. (e) Scheme of ECR-to-CO activity for Ni-SAC@NFC. Reproduced from ref 130. Copyright 2024, American Chemical Society. (f) Scheme of ECR-to-CO activity for Ni–N_4_/C-NH_2_. Reproduced from ref 209. Copyright 2021, Royal Society Chemistry. (g) Optimized structures of Ni–N_4_, V_1_–Ni-N_4_, V_2_–Ni-N_4_, and V_3_–Ni-N_4_. (h) Free energy diagram for the ECR-to-CO conversion. Reproduced from ref 81. Copyright 2025, American Chemical Society. (i) Schematic diagram of the orbital matching mechanism, which originated from the characteristic d-d and d-p orbital interactions between the substrate and the dispersed metal atoms. The ″volcano-type″ scaling relationship between the number of valence electrons (n_ve_) of dispersed elements and (j) single atom (k) cluster formation energies. Reproduced from ref 78. Copyright 2025, Wiley-VCH. (l) Different spin states of Co, Fe, and Mn atoms in TM-TCSACs (TM = Co, Fe, and Mn) and CO*. Reproduced from ref 71. Copyright 2025, American Chemical Society.

Beyond the aforementioned in-plane coordination tuning, out-of-plane (axial) modulation can also break the symmetric electronic structure of M_1_-N_4_ sites, thus optimizing the binding energies of *COOH and *CO. For example, Zhang et al. synthesized axial O-coordinated O–Fe–N-C SASCs using O/N-rich MOFs (IRMOF-3) as precursors. The resulting FeN_4_–O sites exhibited significantly enhanced performance for CO production compared to O-free FeN_4_ sites, reaching an FE_CO_ of 95% at – 0.50 V vs RHE. DFT results indicated that although the *COOH formation barrier for O–Fe–N-C was higher than that for FeN_4_ at both U = 0 V and U = – 0.48 V, the *CO desorption energy was lower, suggesting that the introduction of axial O coordination facilitates CO release and improves ECR-to-CO selectivity (Figure 7d).^208^ In addition to direct coordination tuning, modulating the environment around the metal active sites is another effective strategy to optimize the absorption of key intermediates (e.g., *COOH and *CO) for efficient ECR-to-CO. This includes the introduction of specific functional groups or structural defects, such as C–F bonds, edge-NH_2_ groups, and edge-rich vacancies. For instance, Wang et al. incorporated F atoms into N-doped carbon-supported Ni SASCs via a simple pyrolysis method. The synthesized Ni-SAC@NFC catalyst achieved a FE_CO_ exceeding 99% across a wide potential range and an exceptional CO evolution rate of 9.5 × 10^4^·h^–1^ at −1.16 V vs RHE (Figure 7e).^130^ Similarly, Chen et al. reported a universal amination strategy to significantly boost the current density of M-N/C (M = Ni, Fe, Zn) catalysts for CO production. To be specific, the aminated Ni SASC (Ni–N_4_/C-NH_2_) reached a *j*_CO_ of 450 mA·cm^–2^ at −0.89 V vs RHE, while maintaining a FE_CO_ above 85% over a broad potential window from −0.5 to −1.0 V vs RHE (Figure 7f).^209^ Furthermore, Mei et al. employed support vacancy engineering to introduce edge-rich Ni–N_4_ sites, which enhanced the adsorption of *COOH intermediates. DFT calculations revealed that these edge-rich sites caused an upward shift of the Ni *d*-band center, which strengthened *COOH binding and improved reaction kinetics in acidic media (Figure 7g, h).^81^

The M_1_-M’_*x*_ SASCs, representing a novel alloy design concept, has demonstrated significant performance enhancements by maximizing atomic efficiency, promoting hydrogen spillover, and breaking conventional scaling relations.^210^ Currently, studies on M_1_-M’_*x*_ SASCs for ECR-to-CO are relatively underexplored, likely due to the instability or aggregation of single atoms during synthesis or under harsh reaction conditions. Therefore, Xu et al. proposed a universal screening principle to evaluate the dispersion stability of M_1_-M’_*x*_ combinations based on stabilization energy. Guided by a *d*-*p* orbital matching strategy between the substrate and dispersed atoms, they revealed that guest metals with a *d* ^1^ electronic configuration (or full inner *d*-orbitals with partially occupied outer *s* and *p* orbitals) exhibit superior stability compared to those with only partially occupied *d*-orbitals (Figure 7i). This study also identified the valence electron number (n_ve_) as the most critical intrinsic factor, showing a volcano-type scaling relationship between n_ve_ and formation energy for both single atoms and triatomic clusters (Figure 7j, k). The interesting finding implies that more negative formation energies lead to a higher thermodynamic stability. As a proof of concept, the synthesized Sb_1_Cu catalyst exhibited a FE_CO_ of 99.73 ± 2.5% at 200 mA·cm^–2^ with other synthesized Cu-based single-atom alloys demonstrated over 70% selectivity for C_1_ products.^78^

In contrast to M_1_-N_*x*_ and M_1_-M’_*x*_ SASCs, M_1_-O_*x*_ SASCs are least applied in ECR-to-CO, which may be explained by their inherent electronic rigidity and the difficulty in tuning their coordination environments. Nevertheless, the emergence of spin-state engineering provides a new dimension for optimizing these systems. For example, Liu et al. have found that unlike octahedral compounds (e.g., transition metal oxides), tetrahedral geometry features three high-energy *t*_2g_ orbitals (*d*_*xy*_, *d*_*yz*_, *d*_*xz*_) closer to the Fermi level, offering more opportunities for interaction with reactants and intermediates. Consequently, they synthesized tetrahedral coordination single-atom catalysts (TCSACs) by incorporating 3*d* transition metals (TM = Fe, Mn, Co, etc.) into tetrahedral ZnO sites. The electronic configurations of these TCSACs were elucidated by combining TM oxidation states from X-ray photoelectron spectroscopy (XPS) and magnetic moments from zero-field-cooling (ZFC) measurements. Their findings showed that only Fe and Co maintained intermediate-spin (IS) states before and after CO adsorption (Figure 7l). Notably, Fe, possessing more unpaired electrons, exhibited a moderate binding strength for *CO, which effectively enhanced the CO activation. Experimentally, the Fe-TCSAC achieved a FE_CO_ of 91.6% at – 0.9 V vs RHE and demonstrated excellent stability over 30 h, validating the feasibility of spin-state engineering for efficient ECR-to CO.^71^

In summary, the above research collectively demonstrates that precise modulation of the electronic structure of metal active sites—whether through coordination engineering, peripheral environment tuning, or spin-state control—is the core of optimizing the absorption of key intermediates (e.g., *COOH and *CO). These advancements provide a clear theoretical blueprint and a diverse array of material platforms for designing high-performance SASCs for efficient ECR-to-CO.

#### 4.1.2 CH_4_

CH_4_ stands out for its high combustion efficiency and the highest energy density (∼56 kJ·g^–1^) among all hydrocarbons, making it a critical feedstock in both the energy and chemical industries.^211^ Currently, ECR-to-CH_4_ is primarily dominated by M_1_-N_*x*_ and M_1_-O_*x*_ SASCs, whereas M_1_-M’_*x*_ SASCs are rarely reported. This phenomenon may be attributed to the limited number of unpaired electrons in M_1_-M’_*x*_ sites, which is insufficient to effectively activate *CO and facilitate its continuous hydrogenation to CH_4_. Based on this, the following section mainly focuses on the applications of M_1_-N_*x*_ and M_1_-O_*x*_ SASCs for ECR-to-CH_4_ under various modulation strategies.

Among M_1_-N_*x*_ SASCs, Cu-based catalysts have emerged as the most prominent candidates, likely stems from their high energy barriers for C–C coupling and the favorable protonation of key *CHO intermediates.^216^ Now, various strategies have been proposed to further enhance the ECR-to-CH_4_ activity of Cu–N_*x*_ SASCs. For instance, Li et al. employed a support geometric engineering strategy using N-doped carbon (NC) and graphite sheets (ss) as primary and secondary supports, respectively. By adjusting the coating amount of polydopamine on the ss, they precisely tuned the site spacing (d_site_) of Cu–N_4_ from 2.2 nm (ssCuNC40) to 0.36 nm (ssCuNC160), achieving uniform anchoring of single-atom sites (Figure 8a, b). DFT calculations revealed that reducing d_site_ to 0.68 nm (ssCuNC100) induced a positive shift in the Cu 3*d*_x2‑y2_ orbital, which not only enhanced *CO adsorption but also facilitated the protonation of *OCH_3_ into *CH_4_. As a result, the ss-CuNC100 catalyst exhibited an FE_CH4_ of 70% and a *j*_CH4_ of 303.9 mA·cm^–2^, which is more than 1.5 times higher than that of the unmodified CuNC.^113^ Furthermore, optimizing the adsorption of key intermediates (e.g., *CO and *CHO) on Cu sites can also be achieved through the introduction of a less electronegative atom to conventional coordination N atoms. For instance, DFT calculations by Dai et al. revealed that incorporating a less electronegative B atom (∼2.04) into the Cu–N_4_ coordination environment to form Cu–N_*x*_B_*y*_ significantly strengthens the binding of *CO and *CHO, thereby potentially lowering the energy barrier for CH_4_ formation. Inspired by this, they synthesized BCN-Cu SASCs dominated by Cu–N_2_B_2_ sites. Electrochemical tests showed that this catalyst achieved a FE_CH4_ of 73% at −1.46 V vs RHE and a maximum *j*_CH4_ of −462 mA·cm^–2^ at −1.94 V vs RHE (Figure 8c).^105^

**Figure 8:**
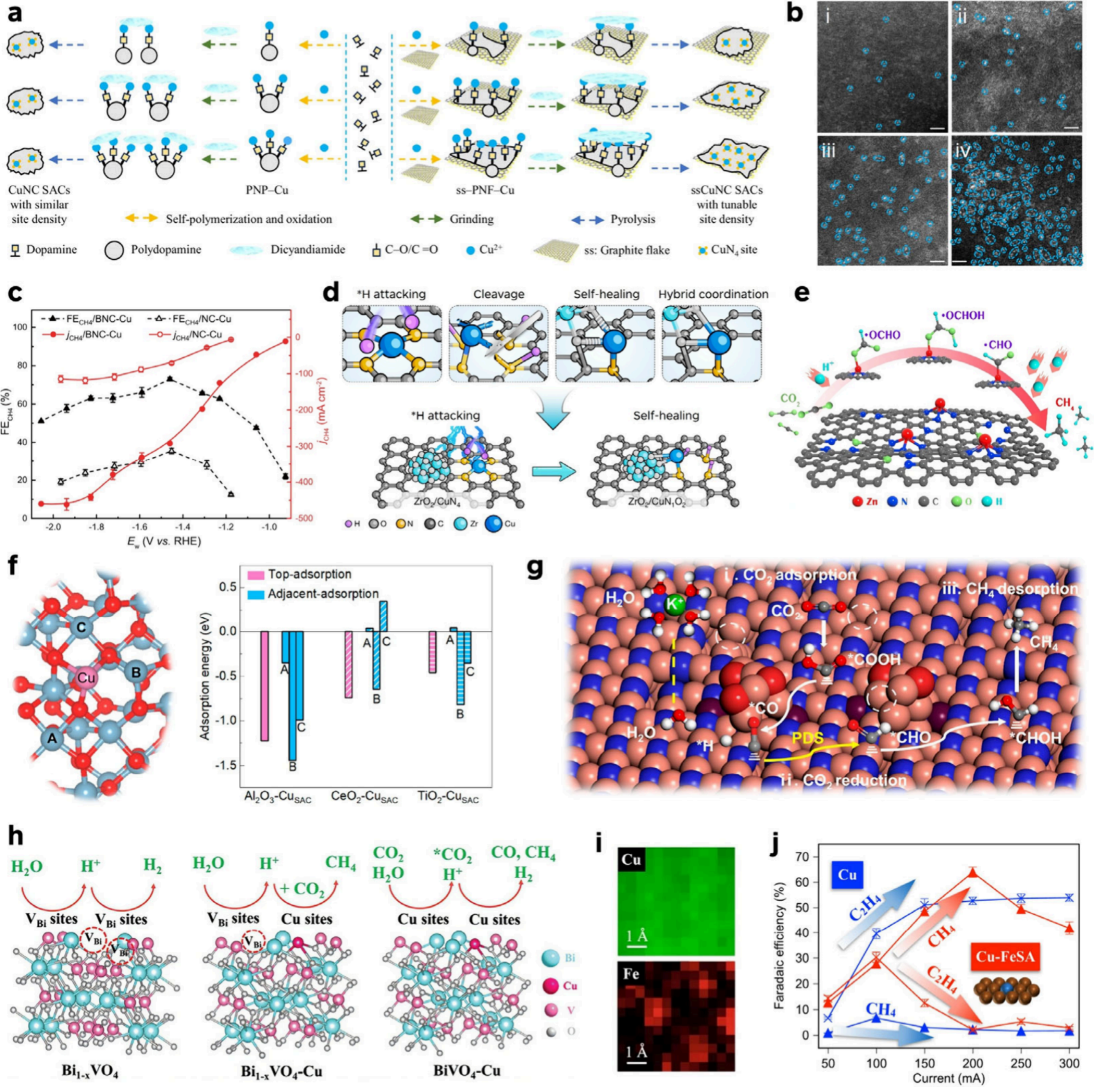
(a) Schematic of the synthesis of CuNC and ssCuNC via support engineering. DCD: Dicyandiamide, PNP-Cu: Cu-chelated polydopamine nanoparticles, ss-PNF-Cu: Cu-bound polydopamine nanoparticles and films coated on ss. (b) AC-HAADF-STEM images of (i) ssCuNC40, (ii) ssCuNC80, (iii) ssCuNC100, and (iv) ssCuNC160. Reproduced from ref 113. Copyright 2025, Springer Nature. (c) FE and partial current densities for CH_4_ of BNC-Cu and NC-Cu as a function of cathodic potentials. Red curve for partial current density and black curve for methane FE. The error bars of FE_CH4_ and *j*_CH4_ are calculated based on three independent measurements. Reproduced from ref 105. Copyright 2023, Springer Nature. (d) Conceptual schematic of the self-healing coordination reconstruction process. Reproduced from ref 212. Copyright 2025, Springer Nature. (e) Scheme of ECR-to-CH_4_ activity for SA-Zn/MNC. Reproduced from ref 213. Copyright 2020, American Chemical Society. (f) Left: Schematic diagram of CO adjacent-adsorption on the Al_2_O_3_–CuSAC surface. Right: CO adsorption energy of Al_2_O_3_–CuSAC, CeO_2_–CuSAC, and TiO_2_–CuSAC. Reproduced from ref 72. Copyright 2025, Springer Nature. (g) Scheme of ECR-toCH_4_ activity for SA-Zn/MNC. Reproduced from ref 214. Copyright 2022, American Chemical Society. (h) Diagrams of the electroreduction processes over Bi_1–*x*_VO_4_, Bi_1–*x*_VO_4_–Cu, and BiVO_4_–Cu. Reproduced from ref 160. Copyright 2023, Wiley-VCH. (i) Atomic elemental mapping using the EELS. (j) Comparison of reaction products for pristine Cu and Cu-FeSA. Error bars represent 1 standard deviation on the basis of three independent samples. Reproduced from ref 215. Copyright 2022, Springer Nature.

Considering that traditional Cu–N_4_ sites are susceptible to adsorbed protons (*H) induced degradation, incorporating more electronegative O atom (∼3.44) to form Cu–O coordination has been proposed as a potential solution to resist *H attack.^217,218^ However, Shen et al. noted that an excessive increase in electronegativity from Cu–O coordination alone might cause an electronic imbalance, failing to resolve the trade-off between activity and stability. Herein, a self-healing strategy enables a one-step Cu–N to Cu–N/O transition, proceeding via HER-induced ″coordination cutting″ and spontaneous O bonding on ZrO_2_/Cu composites (Figure 8d). As a result, compared to pristine Cu–N_4_, the synthesized ZrO_2_/CuN_1_O_2_ SASCs demonstrated a 3-fold increase in FE_CH4_ (87.06% vs 27.8%) at −500 mA·cm^–2^ and a 10-fold increase in FE_CH4_ (80.21% vs 8%) at −1000 mA·cm^–2^. Meanwhile, minimal performance degradation (<3%) after 25 h of operation in a membrane electrode assembly (MEA) electrolyzer further highlighted its exceptional stability.^212^ Parallel to the efforts on Cu SASCs, Zn also demonstrates great potential in the field of ECR-to-CH_4_. As early as 2017, He et al. found via DFT that the overpotential for CH_4_ synthesis on Zn single atoms supported on defective graphene (−0.73 V vs RHE) was lower than that of Cu single atoms (−1 V vs RHE).^219^ Motivated by this, Han et al. developed a Zn single-atom catalyst supported on microporous N-doped carbon (SA-Zn/MNC) for efficient CH_4_ production (Figure 8e). In detail, SA-Zn/MNC exhibited a high FE_CH4_ of 85% at −1.8 V vs SCE, with a corresponding partial current density and yield of −31.8 mA·cm^–2^ and 158 ± 4 μmol·h^–1^·cm^–2^, respectively. DFT calculations indicated that during the ECR process, the O atoms (rather than C atoms) in *OCHO prefer to bond with Zn single atoms, thereby blocking CO evolution and promoting CH_4_ generation.^213^

In the realm of M_1_-O_*x*_ SASCs, Zhang et al. synthesized copper single-atom catalysts supported on Al_2_O_3_ (Al_2_O_3_–CuSAC), CeO_2_ (CeO_2_–CuSAC), and TiO_2_ (TiO_2_–CuSAC) via the ALD method to investigate the mechanism of electronic metal–support interaction (EMSI) in ECR-to-CH_4_. DFT calculations revealed that the electronegativity disparity of Cu (∼1.90) and Ce (∼1.12) allows Cu to withdraw electrons from Ce, markedly reducing electron delocalization around the Cu single atoms. As shown in Figure 8f, the adjacent (site A-C)-adsorption energy of CO (0.35 to −0.65 eV) on the Cu atom of CeO_2_–CuSAC is significantly lower than the top-adsorption energy, leading to lower CO coverage around the Cu sites and consequently hindering the C–C coupling pathway. Furthermore, compared to Al_2_O_3_–CuSAC (1.24 eV) and TiO_2_–CuSAC (−0.52 eV), CeO_2_–CuSAC possesses a moderate free energy for *H_2_O dissociation (−0.24 eV), which facilitates the deep hydrogenation of *CO without excessive activation of the HER. As a result, CeO_2_–CuSAC delivered the highest FE_CH4_ of 70.3% at 400 mA·cm^–2^ among the three catalysts.^72^ In addition, Chen et al. further investigated the H_2_O activation issues highlighted above. To address the inherent limitation of Cu in H_2_O activation due to its high thermal stability,^220^ they employed an Ir single-atom doping strategy to induce nucleophilic attack on H_2_O molecules, thereby accelerating H_2_O dissociation through abundant electrophilic O species. Simultaneously, they integrated an A-site vacant anti-ReO_3_ perovskite-type Cu_3_N with Cu_2_O, which exhibits favorable affinities for *H_2_O and *CO, further optimizing the reaction pathway from *CO to *CHO (Figure 8g). Consequently, the Ir_1_–Cu_3_N/Cu_2_O multiactive site catalyst exhibited a high current density of 320 mA·cm^–2^ at −1.3 V vs RHE, with a maximum FE_CH4_ of 75%.^214^ Meanwhile, Guo et al. utilized Bi vacancies (V_Bi_) to both promote H_2_O dissociation and induce electron transfer toward the Cu single-atom sites in the synthesized Bi_1–*x*_VO_4_–Cu SASCs. This synergistic effect significantly facilitated the formation and stabilization of the key intermediate *CHO, achieving efficient FE_CH4_ of 92% (Figure 8h).^160^

Aside from M_1_-N_*x*_ and M_1_-O_*x*_ SASCs, Hung et al. reported a unique Fe SASC utilizing metallic Cu as the support. By assembling Fe phthalocyanine onto the Cu surface and reducing it during ECR, Fe single atoms were embedded into the Cu host to form Cu-FeSA SASC, which is confirmed by atomic elemental mapping using EELS (Figure 8i). Electrochemical tests demonstrated that Cu-FeSA exhibited a maximum FE_CH4_ of 64% at 200 mA·cm^–2^, which is far superior to the 2% observed for pure Cu (Figure 8j). DFT calculations suggested that Cu-FeSA favors a pathway involving the hydrogenation of *CO to *COH rather than C–C coupling for CH_4_ production.^215^

In general, current research on high-efficiency ECR-to-CH_4_ primarily focuses on the atomic-level precision design and regulation of local coordination environments, support interactions, and interface structures in M_1_-N_*x*_ and M_1_-O_*x*_ SASCs. The purpose is to balance the adsorption of key intermediates (*CO, *CHO/*COH, etc.), proton supply efficiency, and the stability of catalytic sites to achieve high activity, selectivity, and durability. Future efforts could focus on overcoming challenges in the less-explored M_1_-M’_*x*_ SASCs to achieve efficient CH_4_ synthesis. Furthermore, beyond CH_4_, ECR-to-CH_3_OH also represents a high-value research direction due to its extensive applications in the energy and chemical sectors. However, as the overall number of studies on SASCs in the field of ECR-to-CH_3_OH remains limited, it is not discussed in detail in this review.^40,221,222^

#### 4.1.3 HCOOH

HCOOH has long been recognized as a vital liquid feedstock for synthesizing high-value chemicals and an ideal hydrogen carrier, owing to its high volumetric hydrogen storage capacity (53.4 g·L^–1^) and moderate gravimetric capacity (4.4 wt %) under ambient conditions.^223^ Consequently, ECR-to-HCOOH offers a sustainable and efficient pathway for its production. To date, M_1_-N_*x*_, M_1_-M’_*x*_, and M_1_-O_*x*_ SASCs have been extensively developed for the efficient synthesis of HCOOH, with research primarily focusing on main-group metals (e.g., In, Sn, Bi, and Sb) and transition metals (e.g., Cu, Fe, Mo, and Au).

For M_1_-N_*x*_ SASCs, various structural engineering strategies have been employed to enhance HCOOH electrosynthesis such as in-plane or out-of-plane coordination regulation. For the first type, Shang et al. synthesized In-SAs/NC catalysts featuring unique In^δ+^-N_4_ (0 < δ < 3) atomic interface groups on MOF-derived N-doped carbon substrate via a pyrolysis method (Figure 9a). The catalyst achieved a remarkable turnover frequency (TOF) of 12,500 h^–1^ at −0.95 V vs RHE, and a high FE_HCOOH_ of 96% at a lower potential of −0.65 V vs RHE, representing a significant advancement in catalytic performance.^224^ However, such highly symmetric M_1_-N_4_ structures often limit the fine-tuning of the electronic structures of the active metal sites. To overcome this, Dong et al. synthesized a planar-symmetry-broken CuN_3_ (PSB-CuN_3_) catalyst using a microwave-assisted method. DFT calculations revealed that reducing the symmetry from planar-like *D*_*4h*_ (CuN_4_) to *C*_*2v*_ (CuN_3_) configuration caused the antibonding 4s and 4p states to shift significantly upward above the Fermi level (Figure 9b). Furthermore, under an applied electric field (U_RHE_ = −0.8 V vs RHE), the free-energy difference (ΔG_L_) for the rate-determining step (RDS) of HCOOH formation (*OCO → *OCHO) on CuN_3_ was only −0.03 eV, substantially lower than the 0.62 eV observed for CuN_4_, indicating a strong preference for HCOOH production. Experimental results confirmed that PSB-CuN_3_ achieved a maximum FE_HCOOH_ of 94.3% at −0.73 V vs RHE, outperforming the symmetric CuN_4_ catalyst (FE_HCOOH_ = 72.4% at −0.93 V vs RHE).^133^ Besides, Wang et al. introduced S atoms, which have lower electronegativity (∼2.58), into the Fe–N_4_ coordination environment on N-doped carbon supports to form Fe–N_2_S_2_ active sites. This modulation of the electronic structure of the central Fe atom optimized the adsorption of key intermediates (e.g., *CO_2_ and *OCHO). Electron density difference (EDD) maps obtained from DFT calculations showed that the atomic Fe site in Fe–N_2_S_2_ exhibited a charge density higher than that in Fe–N_4_. This suggests that S-substitution breaks the original symmetry of Fe–N_4_ and induces electronic redistribution, correlating with a higher polarization center in Fe–N_2_S_2_ (Figure 9c). Consequently, the Fe–N_2_S_2_ catalyst achieved a FE_HCOOH_ of 90.6% at −0.5 V vs RHE, a stark contrast to the Fe–N_4_ catalyst, which primarily produced CO with a FE_CO_ of 58.7% (Figure 9d).^89^

**Figure 9:**
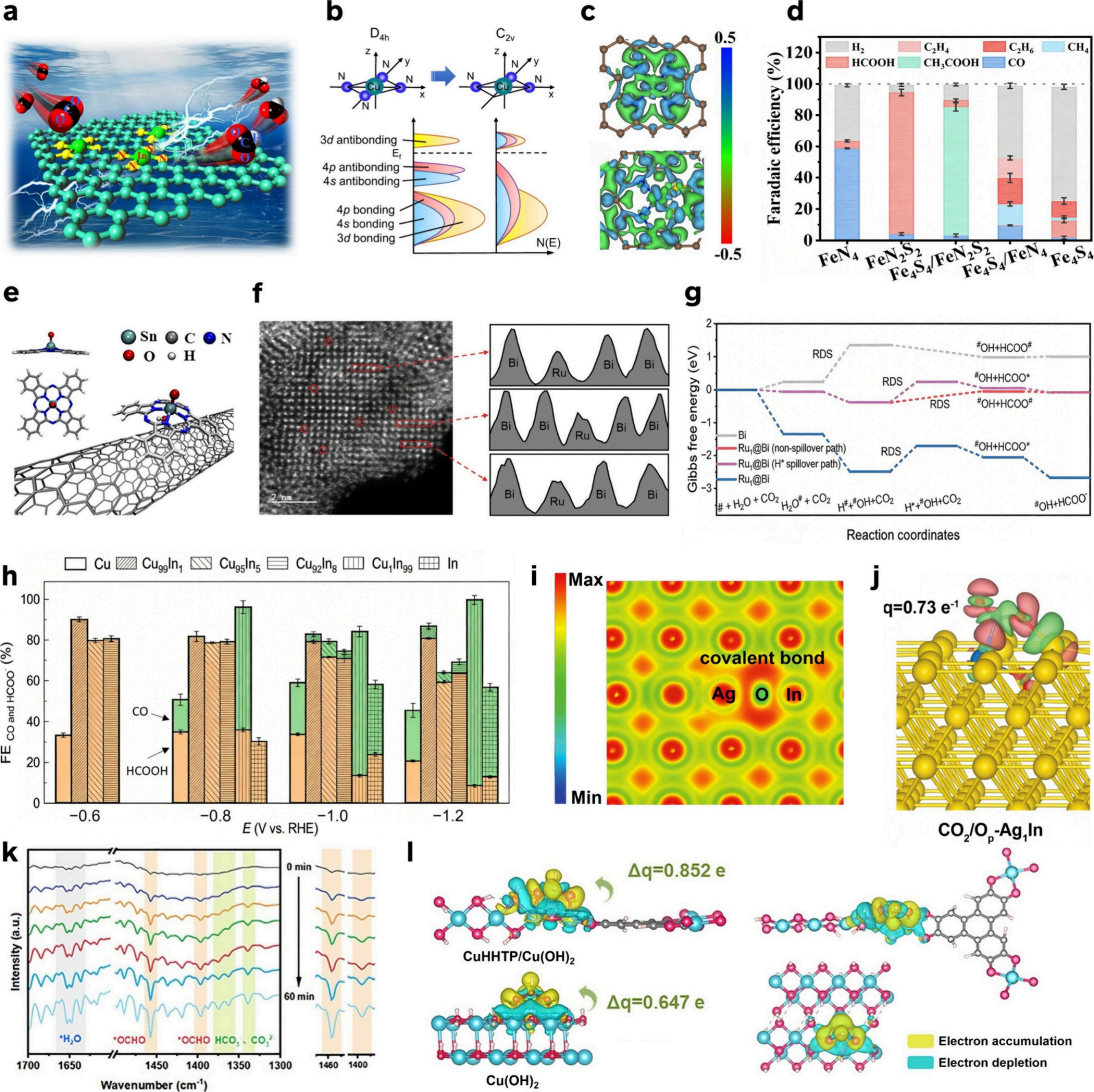
(a) Scheme of ECR-to-HCOOH activity for In Sas/NC. Reproduced from ref 224. Copyright 2020, Wiley-VCH. (b) Schematic diagrams of band shifts and hybridization for the planar CuN_4_ moiety with local *D*_*4h*_ symmetry and the defective CuN_3_ moiety with lower local *C*_*2v*_ symmetry. a.u. arbitrary units. Reproduced from ref 133. Copyright 2023, Springer Nature. (c) EDD plots of FeN_4_ and FeN_2_S_2_. (d) Distribution of ECR products over different Fe-based catalysts at −0.5 V. Reproduced from ref 89. Copyright 2025, American Chemical Society. (e) Schematic illustration for the preparation of SnPc/CNT–OH. Reproduced from ref 128. Copyright 2023, American Chemical Society. (f) Atomic-resolution AC-HAADF-STEM image and intensity profiles of Ru_1_@Bi. (g) Gibbs free energy landscapes for ECR-to-HCOOH at 0 V versus RHE. The RDS is labeled with a black text. Reproduced from ref 143. Copyright 2024, Wiley-VCH. (h) Comparison of FE_CO_ (orange color) and FE_HCOO–_ (green color) on Cu, Cu_99_In_1_, Cu_95_In_5_, Cu_92_In_8_, Cu_1_In_99_ and In catalysts. Reproduced from ref 79. Copyright 2025, Wiley-VCH. (i) Electron localization function maps of Op-Ag_1_In. (j) Charge density difference map of CO_2_ on Op-Ag_1_In. Reproduced from ref 225. Copyright 2024, Wiley-VCH. (k) *In-situ* ATR-FTIR spectra on CuHHTP/Cu­(OH)_2_ at −1.7 V for 60 min in a CO_2_-saturated 0.1 M KHCO_3_–KCl electrode. (l) The charge density difference analysis on CuHHTP/Cu­(OH)_2_ and Cu­(OH)_2_. Reproduced from ref 226. Copyright 2025, Wiley-VCH.

Beyond in-plane strategies, out-of-plane regulation is another effective approach for modulating the electronic structure of SASCs. For example, Deng et al. immobilized tin phthalocyanine (SnPc) onto hydroxylated carbon nanotubes (CNT–OH) through π-π interactions, effectively synthesizing SnPc/CNT–OH with an axially coordinated O structure (O–Sn–N_4_). In contrast, the SnPc/CNT with planar Sn–N_4_ sites was prepared using nonhydroxylated carbon nanotubes (CNT) (Figure 9e). Experimental results indicate that the SnPc/CNT–OH catalyst exhibited an FE_HCOOH_ of 89.4% at −1.0 V vs RHE, surpassing SnPc/CNT (70.6%), with a similar trend observed across other potentials. DFT calculations indicated that the energy barrier for the CO_2_ → *CO_2_ step was −0.58 eV for the O–Sn–N_4_ compared to −0.13 eV for Sn–N_4_, suggesting that the axial O facilitates CO_2_ activation. Moreover, O–Sn–N_4_ showed a lower energy barrier for *HCOOH formation (ΔG = 0.8 eV) than Sn–N_4_ (ΔG = 0.93 eV), leading to enhanced electrocatalytic activity for ECR-to-HCOOH.^128^

For M_1_-M’_*x*_ SASCs for ECR-to-HCOOH, their structures can be categorized based on whether the M and M’ atoms are ″pinned″ by other atoms. In the unpinned type, Liu et al. utilized an electrochemical galvanic method to prepare Ru single atoms on Bi substrate (Ru_1_@Bi, 0.6 wt %) and Ru clusters on Bi substrate (Ru_n_@Bi, 1.2 wt %). The AC-HAADF-STEM image of Ru_1_@Bi is shown in Figure 9f. DFT calculations revealed that Ru_1_@Bi possesses a more balanced capability for the adsorption and activation of H_2_O and CO_2_ compared to Ru_n_@Bi (Figure 9g). Consequently, the reaction activity for CO_2_-to-HCOOH on Ru_1_@Bi was approximately twice that of Ru_n_@Bi, maintaining a high FE_HCOOH_ exceeding 93% within a potential range of −0.8 to −1.2 V vs RHE.^143^ Additionally, Du et al. constructed two SAAs catalysts, Cu_99_In_1_ and Cu_1_In_99_, by inverting the host and guest metals. Regarding Cu_1_In_99_, the neighboring In atoms significantly altered the adsorption properties of Cu, stabilizing the bridge-bonded *CO intermediate and promoting the preferential formation of HCOOH, which reached an FE_HCOOH_ of 91% at −1.2 V (Figure 9h).^79^ In the pinned category, Fang et al. developed an O-pinned silver–indium SAA (Op-Ag_1_In) via a mild electrochemical reduction method. This catalyst exhibited a FE_HCOOH_ of 92.03% and a partial current density of 13 mA cm^–2^ at −0.95 V vs RHE, representing a 1.40-fold increase in selectivity and a 2.23-fold increase in activity compared to pure In catalyst. To elucidate the pinning effect of the O atoms on Ag single atoms, electron localization function (ELF) maps from DFT calculations confirmed the formation of covalent bonds in Op-Ag_1_In (Figure 9i). Furthermore, the introduction of O-pinning facilitated the adsorption of *CO_2_ and *H toward HCOOH formation and reduced the kinetic energy barrier of the RDS (Figure 9j).^225^

Finally, catalysts featuring M_1_-O_*x*_ sites constitute a promising frontier for ECR-to-HCOOH, demonstrating significant potential due to their atomically dispersed active centers. Shao et al. strategically anchored phenolic 2,3,6,7,10,11-hexahydroxytriphenylene (HHTP) onto Cu­(OH)_2_ surfaces to construct a CuHHTP/Cu­(OH)_2_ catalyst. The surface phenol-to-quinone transformation regulated the CO_2_ adsorption configuration, creating a unique reaction pathway for efficient HCOOH production. The optimized CuHHTP/Cu­(OH)_2_ has achieved a high current density of 65.6 mA cm^–2^ and a FE_HCOOH_ of 88.8% at −1.4 V vs RHE, maintaining FE_HCOOH_ of 83.0% after 66 h of continuous ECR testing. As shown in Figure 9k, *in situ* electrochemical attenuated total reflection Fourier transform infrared spectroscopy (ATR-FTIR) for CuHHTP/Cu­(OH)_2_ revealed O–C–O stretching vibrations at 1380 and 1446 cm^–1^ corresponding to the *OCHO intermediate. In contrast, peaks associated with the *COOH intermediate were absent, indicating the effective suppression of the competing *COOH pathway. DFT calculations further showed that CuHHTP/Cu­(OH)_2_ exhibits stronger electronic interactions with CO_2_ (Δq = 0.852 e) compared to Cu­(OH)_2_ (Δq = 0.674 e), facilitating electron transfer from Cu to CO_2_ for stable binding and subsequent reduction (Figure 9l). The calculated free energy barriers for CO_2_ conversion to *OCHO were 0.67 eV for CuHHTP/Cu­(OH)_2_ and 0.82 eV for Cu­(OH)_2_, confirming that the ECR-to-HCOOH is more energetically favorable on the former.^226^

In summary, M_1_-N_*x*_, M_1_-M’x, and M_1_-O_*x*_ SASCs have significantly enhanced the selectivity and efficiency of HCOOH production by precisely tailoring the local coordination environment (e.g., symmetry, ligand species, and axial modification) to optimize the adsorption behavior of key intermediates (such as *CO, *HCOO, and *OCHO). Future research can focus on unraveling the dynamic evolution mechanisms of active sites to drive further breakthroughs in catalytic performance, ultimately advancing ECR-to-HCOOH toward high-efficiency, stability, and industrialization.

### 4.2 CO_2_ to C_2+_ Products

As shown in Figure 6, compared to C_1_ products (e.g., CO, CH_4_, and HCOOH), the reaction mechanisms for C_2+_ products (e.g., C_2_H_4_, CH_3_CH_2_OH, CH_3_COOH, and CH_3_COCH_3_) are significantly more intricate, typically involving multistep PCET processes. But it is now a wide consensus in the community that *CO serves as the pivotal intermediate for the formation of C_2+_ species. To date, Cu-based catalysts remain the main class of materials recognized for their ability to efficiently electroreduce CO_2_ to C_2+_ products, a property primarily attributed to their moderate binding energy for *CO. However, research into Cu-based SASCs for this specific application remains relatively limited, largely due to the inherent physical isolation of single-atom active sites, which is unfavorable for the C–C coupling step. Consequently, this section will not categorize discussions by individual C_2+_ products but instead will treat them as a collective whole to provide a systematic and comprehensive overview of the underlying mechanisms and recent advancements.

Currently, research on M_1_-N_*x*_ SASCs for ECR-to-C_2+_ remains the most extensive, with Cu atoms predominantly serving as active centers. Nevertheless, many Cu-based SACs undergo *in situ* reconstruction under operating conditions, evolving from isolated Cu sites into Cu clusters or nanoparticles to facilitate C–C coupling, complicating the elucidation of the structure–activity relationships. To address this, Choi et al. elegantly utilized *operando* high-energy resolution fluorescence detected X-ray spectroscopy (HERFD-XAS) and *operando* electrochemical liquid-cell scanning transmission electron microscopy (EC-STEM) to quantitatively track the structural and molecular evolution from single atoms to nanocrystals. As illustrated in Figure 10a, the *operando* EC-STEM images clearly reveal the transformation of a Cu single-atom into metallic Cu nanoparticles on the carbon support during a 120 s ECR process. Compared to Cu-SAC containing less than half the metallic Cu nanocrystals, the nanocarbon supported Cu-SAC (Cu-SAC-NC), comprising almost entirely metallic Cu nanocrystals, achieved a 5-fold increase in the FE_C2+_. This enhancement was attributed to the superior electronic conductivity of the nanocarbon, which promoted the formation of dense Cu carbonyl (Cu-CO) intermediates, as detected by *in situ* attenuated total reflection surface enhanced infrared absorption spectroscopy (ATR-SEIRAS). Thus, a higher proportion of active metallic Cu nanocrystallites were formed for efficient C–C coupling and significantly improved C_2+_ selectivity.^80^ However, the specific local coordination environments that trigger Cu reconstruction and their subsequent impact on the ECR-to-C_2+_ activity remain poorly understood. To clarify this, Lu et al. selected five well-defined mononuclear copper complexes with distinct ligand structures: Cu­(II) 5,10,15,20-tetraphenyl-21H,23H-porphine (CuTPP), Cu­(II) phthalocyanine (CuPc), fluoro-substituted CuPc (CuPc-F), amino-substituted CuPc (CuPc-NH_2_), and Cu­(II) bis­[1-(2-pyridylazo)-2-naphtholato] (CuPAN). *Operando* XAFS revealed that the Cu–N_4_ in CuPc is more stable than the Cu–N_2_O_2_ in CuPAN, likely due to the easily dissociable Cu–O bonds of the tetrahedral Cu–N_2_O_2_ configuration. Furthermore, compared to other model compounds, only CuPc-F exhibited a transition from Cu­(II) to Cu­(I) during the ECR process, suggesting that the electron-withdrawing F-substituents stabilized the Cu­(I) oxidation state (Figure 10b). Interestinglythe unique Cu­(I) active site facilitates C_2+_ production via a distinctive Cu­(I)­N_3_H-*CO intermediate, which acts as a bridge for *CO transfer to adjacent Cu(0) sites, achieving a total FE for C_2_H_4_ and C_2_H_5_OH of approximately 36% at −1.07 V vs RHE (Figure 10c).^82^

**Figure 10:**
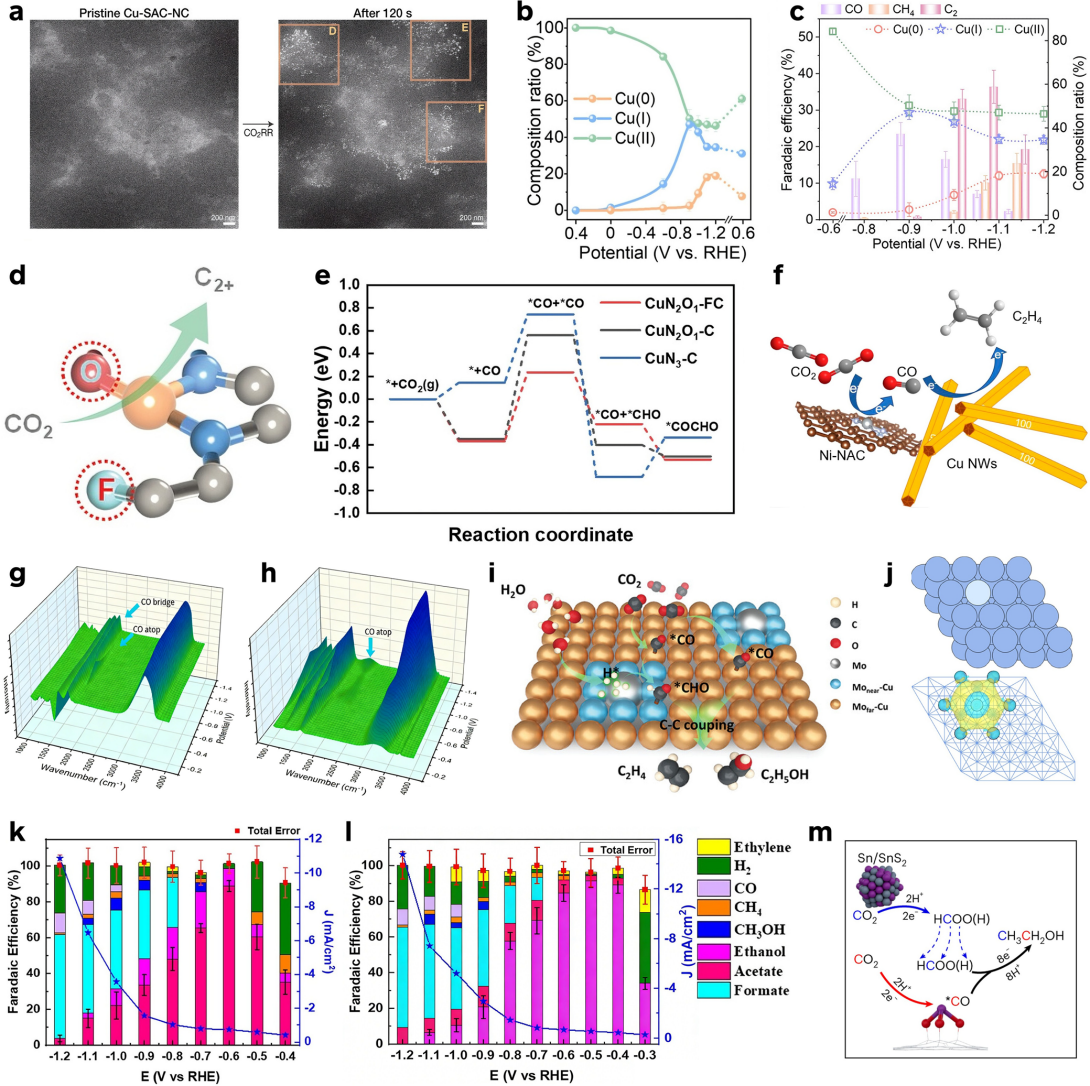
(a) Low magnification EC-STEM images of pristine-SAC and Cu nanograins after the CO_2_RR for 120 s in liquids. Reproduced from ref 80. Copyright 2025, American Chemical Society. (b) Linear combination fitting of Cu oxidation states for CuPc-F. (c) Cu composition ratio and FEs of various products at multiple potentials for CuPc-F. Reproduced from ref 82. Copyright 2025, American Chemical Society. (d) F, O-codrived local coordination environment optimized for CuFONC favors the CO_2_RR toward C_2+_. (e) The reaction pathways of CO_2_ to *COCHO on CuN_2_O_1_–FC, CuN_2_O_1_–C, and CuN_3_–C. Reproduced from ref 121. Copyright 2024, Wiley-VCH. (f) Schematic illustration of the modular design of the Cu/Ni-NAC hybrid catalyst for tandem catalysis. *In-situ* SEIRAS spectra of (g) the Cu/Ni-NAC and (h) the Cu NW catalysts under ECR conditions. Reproduced from ref 95. Copyright 2022, American Chemical Society. (i) A proposed reaction mechanism for the CO_2_RR to C_2_H_4_ and C_2_H_5_OH. Reproduced from ref 144. Copyright 2025, Wiley-VCH. (j) Simulated geometry and charge density difference of M_1_/Cu­(111). Reproduced from ref 68. Copyright 2025, Springer Nature. FEs and the product distributions under different polarization potentials over (k) Sn/C-0.12 and (l) Sn/C-1.2 (stacked bar chart) and the geometric partial current densities, J, toward (k) acetate and (l) ethanol (blue line-stars). All FEs are calculated from chronoamperometry measurements. Reproduced from ref 161. Copyright 2024, American Chemical Society. (m) A schematic illustration showing the cascade reaction during CO_2_ reduction to ethanol over SnS_2_/Sn_1_–O_3_G (gray: S, red: O, yellow: H and purple: Sn). Reproduced from ref 157. Copyright 2023, Springer Nature.

In addition to studies on Cu-SACs prone to *in situ* reconstruction, Lv et al. took an alternative approach by developing a highly stable, high-density Cu single-atom catalyst supported on heteroatom-doped carbon (CuFONC) to reveal definitive structure–activity relationships (Figure 10d). The CuFONC catalyst, featuring a high Cu loading (21.9 wt %) and a stable CuN_2_O_1_–FC configuration, exhibited an exceptional FE_C2+_ of ∼80.5% at −1.3 V vs RHE. DFT calculations indicated that CuN_2_O_1_–FC possesses the lowest binding energy (−4.19 eV) compared to those of CuN_3_–C (−2.76 eV) and CuN_2_O_1_–C (−4.33 eV), suggesting that the introduction of F and O atoms enhances catalyst stability (Figure 10e). Moreover, the CuN_2_O_1_–FC configuration showed the lowest reaction energy barriers for CO_2_ to *CO and *CO to *CO + *CO, indicating that F and O atoms also synergistically optimize the adsorption of key intermediates, thereby significantly boosting ECR-to-C_2+_ performance.^121^

Given that *CO is the critical intermediate for C–C coupling, tandem strategies, where one catalyst synthesizes *CO and another facilitates C–C coupling, represent a promising route for efficient ECR-to-C_2+_ products. For instance, Yin et al. reported a hybrid catalyst integrating Ni single atoms with Cu nanowires for efficient C_2_H_4_ synthesis (Figure 10f). The Ni single-atom catalyst on a N-doped carbon support (Ni-NAC) achieved a FE of over 90% for CO_2_-to-CO conversion over a wide potential range. Therefore, by hybridizing Ni-NAC with Cu nanowires (dominated by {100} facets favorable for C–C coupling) to form Cu/Ni-NAC, this hybrid catalyst achieved an FE_C2H4_ of 66% in an alkaline flow cell with a current density exceeding 100 mA·cm^–2^ at – 0.5 V vs RHE. As shown in Figure 10g, h, the hybrid Cu/Ni-NAC catalyst exhibited two strong CO adsorption peaks at 2050 and 1950 cm^–1^, clearly indicating that *CO generated by the Ni-NAC module was transferred to and enriched on the Cu nanowire surface, providing direct evidence for subsequent C–C coupling.^95^

In contrast to M_1_-N_*x*_ SACs with relatively isolated active sites, M_1_-M’_*x*_ SASCs allow for the construction of multiactive sites by doping a second metal atom into another metal substrate, which is more conducive to ECR-to-C_2+_ requiring C–C coupling. For example, Jin et al. reported a Mo_1_Cu SAA catalyst that achieved a C_2+_ partial current density of 1.33 A·cm^–2^ with an FE exceeding 74.3% (Figure 10i). DFT calculations suggested that the introduced Mo sites promote H_2_O dissociation to generate active *H, while Cu^0^ sites distal to Mo act as active centers for CO_2_ activation to CO. Furthermore, CO and *H are captured by adjacent Cu sites (Cu^&+^) near the Mo atoms, accelerating CO conversion and the C–C coupling process.^144^ Similarly, Huang et al. utilized a single-atom In-alloyed Cu (In_1_/Cu) catalyst in a high-pressure MEA to convert gas-phase CO_2_ to C_2_H_4_, achieving a maximum FE_C2H4_ of 85% and a current density of 750 mA·cm^–2^ under 20 bar (Figure 10j).^68^

Beyond M_1_-N_*x*_ and M_1_-M’_*x*_ SASCs, a limited number of M_1_-O_*x*_ SACs have also been explored for C_2+_ production. For instance, Xu et al. reported a series of carbon-supported Sn electrocatalysts with Sn sizes ranging from single atoms and ultrasmall clusters to nanocrystals (Sn/C-0.12, Sn/C-1.2, and Sn/C-12). By tuning the size of the Sn active sites, the reaction pathway for ECR-to-C_2+_, including CH_3_COOH and C_2_H_5_OH, has been controlled with high selectivity (FE > 90%) and low onset potentials. Specifically, Sn/C-0.12 converted CO_2_ to CH_3_COOH with 90% FE (at −0.6 V), while Sn/C-1.2 yielded C_2_H_5_OH with 92% FE (at −0.4 V) (Figure 10k, l).^161^ Additionally, Ding et al. reported a Sn-based tandem electrocatalyst for ECR-to-C_2_H_5_OH conversion, comprising SnS_2_ nanosheets and single Sn atoms anchored on a 3D carbon support via local Sn–O_3_ clusters (Sn_1_–O_3_G). They found that SnS_2_ nanosheets produce HCOOH intermediates, while Sn_1_–O_3_G sites generate *OCO­(OH) bicarbonate intermediates, followed by C–C coupling at active centers composed of Sn and O atoms. This strategy enabled an ethanol selectivity exceeding 70% over a broad potential range of −0.6 to −1.1 V vs RHE, maintaining 97% FE at current densities up to 17.8 mA·cm^–2^ of its initial activity after 100 h of continuous operation (Figure 10m).^157^

Overall, the scientific community has developed numerous strategies to tune SASCs to meet the requirements for efficient ECR-to-C_2+_ conversion. However, constrained by the physical isolation of active sites in conventional SASCs, recent reports have begun to explore dual-atom site catalysts (DASCs) and even multiatom site catalysts with multiple active sites, though these studies remain relatively scarce.^227^ Therefore, further in-depth exploration in this direction would be needed to meet the demands for the high-efficiency synthesis of C_2+_ products.

## 5 Conclusions and Outlook

In summary, SASCs, characterized by maximized atom-utilization efficiency and well-defined active sites, serve as an ideal model system to break traditional linear scaling relationships and achieve precise product selectivity in ECR. This stands in contrast to conventional nanoparticle catalysts, which offer controllable size and morphology as well as notable stability but whose activity is often governed by surface sites with low atomic utilization, and to molecular catalysts, which offer structural precision but face challenges in stability and device integration. In this review, we systematically summarize the precise synthesis strategies for the three main SASCs types: M_1_-N_*x*_, M_1_-M’_*x*_, and M_1_-O_*x*_. Subsequently, we highlighted the historical evolution and advantages of AC-HAADF-STEM, XAFS, and Mössbauer spectroscopy in probing coordination structures of SASCs. Crucially, we underscored the intrinsic limitations of these advanced techniques, advocating that researchers must adopt a ″multitechnique combination″ strategy to cross-validate findings and overcome the bottlenecks associated with individual methods. Last, we have elucidated the specific structure–activity relationships linking diverse coordination environments to the high selectivity of target C_1_ and C_2+_ products. However, despite the remarkable progress in this field, the transition toward large-scale practical application remains fraught with challenges. To address these hurdles, we propose the following potential directions for future research:

**1. Tuning for high-selectivity C_2+_ products:** In the field of ECR, *CO is widely recognized as the key intermediate for C_2+_ product synthesis via C–C coupling. While Cu SASCs possess more suitable *CO adsorption energies than their CO-selective counterparts (e.g., Ag, Ni, Fe, and Mn SASCs), they still struggle to achieve high C_2+_ selectivity due to the physical isolation of their active sites, which impedes C–C coupling. Therefore, two main strategies have been proposed to overcome this hurdle. The first is the design of multiatom catalysts (e.g., dual-, triple-, or multiatom sites). These catalysts utilize adjacent active sites to coactivate *CO or adsorb various intermediates, thereby lowering the C–C coupling barrier for C_2+_ products like ethanol and 1-propanol. However, as discussed in Section 3.4, the structural characterization of such multiatom centers is challenging due to the limitations of AC-HAADF-STEM, XAFS, and Mössbauer spectroscopy, making it difficult to establish clear structure–activity relationships. The second strategy entails constructing tandem systems that combine CO-selective SASCs with Cu SASCs to facilitate the sequential conversion of CO_2_ to CO and subsequently to C_2+_ products. Currently, tandem catalysis is considered more promising for practical applications as it reduces design complexity while offering superior controllability and predictability.

**2. Unveiling the true dynamic active sites:** To date, most SASCs research in the field of ECR regards the pristine coordination structure (e.g., M_1_-N_4_) as the unchanging active site. However, under the dynamic electrochemical environment, the initial structure may undergo significant reconstruction. For example, Wei et al. synthesized a Zn-SA/CNCl-1000 catalyst via NaCl-assisted pyrolysis and utilized *in situ* FT-EXAFS to monitor its structural evolution during the process of ECR. They observed that under an applied potential of −0.9 V vs RHE, the Zn–N coordination number decreased from 4 at open circuit voltage (OCV) to approximately 3, whereas the Cl-free Zn-SA/CN-1000 catalyst remained unchanged. DFT calculations revealed that the adjacent C–Cl bond, due to the large electronegativity difference between Cl and N, promotes protonation of the coordinating N atom, triggering dynamic evolution from Zn–N_4_ to Zn–N_3_. Consequently, this *in situ* generated Zn–N_3_ site exhibits a lower energy barrier, delivering a CO partial current density of 271.7 mA·cm^–2^ with an FE of 97.5%.^109^ Inspired by such insights, advanced *in situ*/*operando* characterization techniques (e.g., *in situ* EXAFS, *in situ* XAS, and *in situ* Raman spectroscopy) can accurately unveil the true active sites of SASCs under working conditions. This capability allows performance enhancements to be correctly attributed to specific coordination structures, thereby providing instructive insights rather than misleading other researchers into pursuing further studies based on erroneous foundations. Such efforts will provide a reliable experimental basis for the next generation of efficient SASCs design.

**3. Unlocking the rising electronic spin dimension:** In addition to the above-mentioned aspects, manipulating the electronic spin state of the central metal atom in SASCs represents a frontier and transformative new dimension beyond optimizing geometric coordination and charge density. Based on crystal field theory (CFT), the like-charge repulsion between transition metal *d*-orbital electrons and nonbonding electrons of ligand atoms induces the splitting of *d*-orbital energy levels (e.g., *t*_*2g*_ and *e*_*g*_ orbitals in an octahedral field), which directly dictates the electronic spin state.^228^ In brief, when the ligand field splitting energy is smaller than the electron pairing energy, the central metal of SASCs will form a high-spin (HS) state; conversely, it adopts a low-spin (LS) state.^229^ This theoretical understanding enables deliberate spin-state engineering for investigating its potential impact on SASCs catalysts. For instance, Zeng et al. introduced a strong-field-O atom axially to the Fe–N_4_ active site, increasing the ligand field splitting energy and facilitating the formation of low-spin Fe­(III) sites. At a cathodic potential of −0.7 V vs RHE, this catalyst achieved a CO Faradaic efficiency of up to 99% and an exceptionally high turnover frequency (TOF) of 5.3 × 10^4^·h^–1^, representing a performance improvement of over 20-fold compared to high-spin Fe­(III) sites.^230^ In addition to the aforementioned internal regulation strategies, external field regulation (including magnetic, electric, and light field modulation) serves as an innovative strategy to achieve precise control over catalytic performance through noncontact physical interactions, deserving deeper exploration in the field of ECR. Meanwhile, it is imperative to develop and integrate high-sensitivity *in situ* spin-sensitive characterization techniques, such as *in situ* X-ray magnetic circular dichroism (XMCD),^231^ *in situ* electron paramagnetic resonance (EPR), and *in situ* Mössbauer spectroscopy. The application of these methods is essential to precisely capture the dynamic evolution of spin states under reaction conditions, thereby establishing a clear spin-activity relationship of SASCs.

**4. Advancing AI-driven full-lifecycle development:** With the increasing demand for efficiency and precision in SASCs development, the traditional ″trial-and-error″ paradigm has become insufficient to navigate the vast coordination space of SASCs for precise synthesis of high-value ECR products. Artificial intelligence (AI) is leading a paradigm shift from ″passive discovery″ to ″proactive design″, aiming to achieve the full-lifecycle development of SASCs, which covers theoretical prediction, precision synthesis, and performance evaluation. For instance, Bai et al. employed AI for large-scale literature embedding analysis and deep model screening to extract key design features (e.g., magnetic metal centers such as Fe/Co and heteroatom coordination structures such as M-N/S). Then, they constructed binary catalytic descriptors by integrating *d*-band centers and adsorption Gibbs free energy (ΔG) from DFT calculations, enabling the precise and efficient AI screening of high-performance SASCs.^232^ Furthermore, the ″AI-driven automated laboratory″ represents a highly promising frontier for future ECR studies. Szymanski et al. developed an A-Lab platform, which integrates computational simulations, historical literature data, machine learning (ML), and active learning to autonomously plan and execute robotic experiments. During 17 days of continuous operation, A-Lab successfully synthesized 41 new compounds from 58 targets, including various oxides and phosphates.^233^ Based on this, integrating such platforms with *operando* characterization and electrochemical testing feedback loops would establish a closed-loop automated optimization process that encompasses SASCs construction, structural verification, and performance testing. Such a data-driven paradigm will drastically shorten development timelines and facilitate the commercialization of SASCs, effectively bridging the gap between laboratory-scale discovery and industrial-scale implementation.
